# Contributions of conserved and species-specific CagX (VirB9) domains to the assembly and function of the *Helicobacter pylori* Cag type IV secretion system

**DOI:** 10.1128/iai.00699-25

**Published:** 2026-06-10

**Authors:** Chiamaka D. Okoye, Arwen E. Frick-Cheng, Wilhelm Salmen, Sirena C. Tran, W. Hayes McDonald, John T. Loh, Mark S. McClain, D. Borden Lacy, Melanie D. Ohi, Timothy L. Cover

**Affiliations:** 1 Department of Pathology, Microbiology, and Immunology, Vanderbilt University Medical Center 12328, Nashville, Tennessee, USA; 2 Life Sciences Institute, University of Michigan 1259, Ann Arbor, Michigan, USA; 3 Mass Spectrometry Research Center, Vanderbilt University School of Medicine 12327 , Nashville, Tennessee, USA; 4 Department of Biochemistry, Vanderbilt University 5718, Nashville, Tennessee, USA; 5 Department of Medicine, Vanderbilt University School of Medicine 12327 , Nashville, Tennessee, USA; 6 Vanderbilt Institute for Infection, Immunology, and Inflammation, Vanderbilt University Medical Center, Nashville, Tennessee, USA; 7 Veterans Affairs Tennessee Valley Healthcare System, Nashville, Tennessee, USA; 8 Department of Cell and Developmental Biology, University of Michigan 1259, Ann Arbor, Michigan, USA; University of Pennsylvania School of Veterinary Medicine, Philadelphia, Pennsylvania, USA

**Keywords:** *Helicobacter pylori*, bacterial protein secretion, bacterial secretion systems, proteomics, crosslinking mass spectrometry

## Abstract

The *Helicobacter pylori* Cag Type IV Secretion System (T4SS) and the secreted CagA effector protein have key roles in gastric cancer pathogenesis. The Cag T4SS outer membrane core complex (OMCC) consists of an outer membrane cap (OMC), a periplasmic ring (PR), and a stalk. CagX and CagY span both the OMC (14 copies) and PR (17 copies) subassemblies of the OMCC. CagX is structurally related to VirB9 proteins found in other bacterial T4SSs, except for a unique 130-amino-acid region, designated as the periplasmic insertion (PI) domain. In this study, we investigated the contributions of individual CagX domains to Cag T4SS activity and OMCC assembly by engineering *H. pylori* strains that produce only the CagX OMC domain, only the CagX PR domain, the CagX OMC and PR domains produced as independent proteins (Split CagX), or a CagX protein lacking the PI domain (CagX Δ131–260). We found that the OMC, PR, and PI domains of CagX are each essential for Cag T4SS activity. Electron microscopy analyses revealed that the Split CagX mutant could assemble the Cag T4SS PR (17 copies of CagX and CagY), and the CagX Δ131–260 mutant could assemble partially intact OMCCs. Crosslinking mass spectrometry analysis showed that the CagX Δ131–260 protein retained spatial relationships with the OMCC components CagY and CagM. An intact CagX protein was required for the association of CagW, CagH, CagI, and CagL with the OMCC. These results provide novel insights into the contributions of individual CagX domains to the assembly and activity of the Cag T4SS.

## INTRODUCTION

*Helicobacter pylori* is a bacterium that inhabits the stomachs of approximately 40%–50% of the human population (1). While most colonized individuals remain asymptomatic, *H. pylori* is the strongest known risk factor for stomach cancer (2–6), which is a leading cause of global cancer-related deaths (7). Strains of *H. pylori* that harbor the *cag* pathogenicity island (*cag* PAI), a 40-kilobase chromosomal region, have been linked to an increased risk for the development of stomach cancer and peptic ulcer disease (2, 3, 5). The *cag* PAI encodes components of the Cag type IV secretion system (T4SS), a bacterial nanomachine that spans both the inner and outer membranes, as well as CagA, a secreted effector protein (8–14).

Following Cag T4SS-mediated delivery of CagA into gastric epithelial cells, CagA interacts with multiple host cell proteins and can undergo tyrosine phosphorylation (15–20), leading to disruptions in multiple cellular signaling pathways (19, 21, 22). Both human epidemiologic studies and experimental studies using animal models indicate that CagA contributes to the development of gastric cancer, and CagA has been designated as a bacterial oncoprotein (23–29). The Cag T4SS is also required for the delivery of *H. pylori* DNA (30), peptidoglycan (31), and lipopolysaccharide (LPS) metabolites (32, 33) into gastric epithelial cells (3, 8). Cag T4SS-mediated delivery of LPS metabolites (ADP-heptose) and peptidoglycan into host cells leads to the activation of NF-kB and increased production of the proinflammatory cytokine interleukin-8 (IL-8) (34–39), and Cag T4SS-mediated delivery of DNA into host cells leads to the activation of Toll-like receptor-9 (TLR-9) (30, 39, 40).

**Fig 1:**
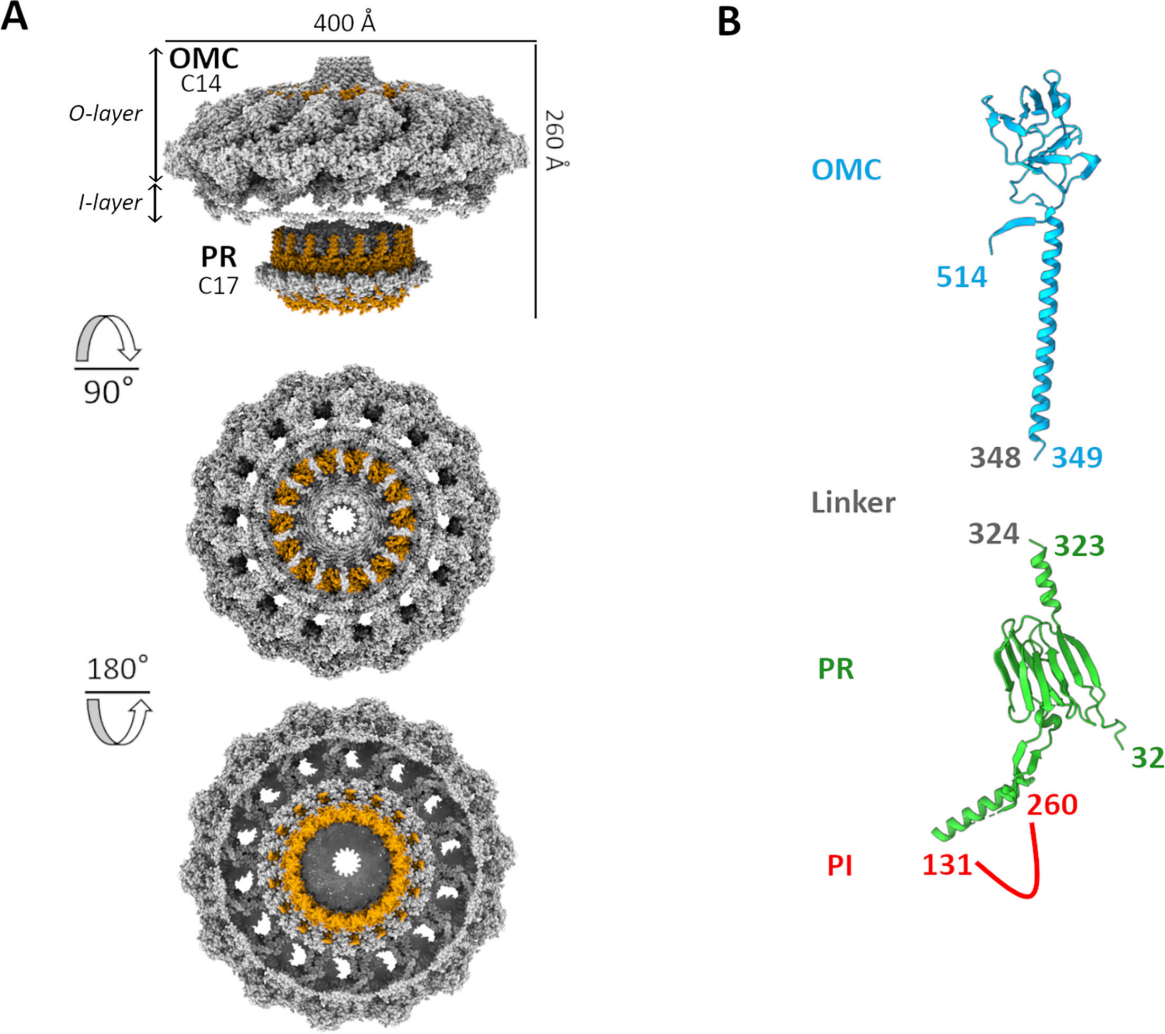
Structure of *H. pylori* CagX, the VirB9 homolog of the Cag T4SS. (**A**) CagX is highlighted in orange within the *H. pylori* Cag T4SS OMCC (PDB: 6X6S and 6X6J) (12). The top image shows the side view, the middle image shows the OMCC rotated 90˚ (view from the outer to inner membrane), and the lower image is rotated 180˚ (view from the inner to outer membrane). (**B**) Structure of one asymmetric unit of CagX, showing its two conserved domains, the outer membrane cap (OMC; blue) and periplasmic ring (PR; green), and an unresolved linker region (amino acids 324–348). The site of an *H. pylori*-specific periplasmic insertion (PI; red) domain is shown as a loop. Densities for the CagX linker region and PI domain were not visible in the cryo-EM density maps.

Structural insight into the organization of the Cag T4SS has been attained using multiple electron microscopy (EM) approaches, including visualization of the Cag T4SS in vitrified *H. pylori* cells by cryo-electron tomography (11, 41) and single-particle cryo-EM analysis of isolated Cag T4SS outer membrane core complexes (OMCCs) (9, 10, 12). Collectively, these studies reveal that wild-type (WT) Cag T4SS OMCCs have a 42 nm *en face* diameter*,* with a characteristic appearance of a wheel with 14 spokes (Fig. 1A) (9). Side views of Cag T4SS OMCCs have been likened to “mushrooms” (9), and single-particle cryo-EM studies have revealed that the OMCC is organized into three subassemblies, designated as an outer membrane cap (OMC), a periplasmic ring (PR), and an unresolved stalk (10, 12).

Subassemblies of the Cag T4SS OMCC are characterized by symmetry mismatches. The OMC is 14-fold symmetric and consists of five proteins (CagY, CagX, CagT, CagM, and Cag3) in a stoichiometric ratio of 1:1:2:2:5, respectively, within each asymmetric unit (12). The PR is 17-fold symmetric and consists of two proteins (CagX and CagY) in a 1:1 ratio within a single asymmetric unit. Fourteen copies of CagX and CagY in the PR extend into the OMC subassembly, and three copies of CagX and CagY in the PR do not extend into the OMC subassembly, creating a symmetry mismatch (12). Both CagX and CagY have a flexible linker region that connects the parts of the proteins found in the OMC and PR (12). Similar symmetry mismatches have been observed in other bacterial T4SSs (42, 43) (Fig. S1A), as well as bacterial flagellar assemblies (44–46), T3SSs, T6SSs, and F_0_F_1_ ATP synthases (47–49). The symmetry mismatch of the *Escherichia coli* R388 and F plasmid T4SS OMCCs has been proposed to contribute to dynamic structural changes that facilitate assembly and function of the associated pilus structures (43, 50). However, the functional contributions of these mismatched symmetries in bacterial secretion systems to the mechanisms of substrate selection and secretion remain elusive.

In addition to the five major components of the Cag T4SS OMCC, four additional proteins—CagW, CagH, CagI, and CagL—co-purify with the OMCC (51). These four proteins are required for activity of the Cag T4SS (51–54) but are present in relatively lower abundance than other OMCC components (51). The localization of these four proteins relative to known structural components of the OMCC is unknown.

When compared with T4SSs from other bacteria, the *H. pylori* Cag T4SS is classified as an expanded T4SS (10, 12), along with the *Legionella pneumophila* Dot/Icm T4SS (55–57), based on its large size and large number of components (42, 43). Prototypical T4SSs encoded by the *E. coli* R388 plasmid (50, 58) and by *Xanthomonas citri* (59) consist of only 12 components, named VirB1-11 and VirD4 (42, 43, 60) (Fig. S1). Three of the five major components of the Cag T4SS OMCC exhibit structural relatedness to VirB proteins of other bacterial T4SSs: CagY (VirB10), CagX (VirB9), and CagT (VirB7) (3, 10, 12, 61). The other two components (CagM and CagT) are species-specific components of the *H. pylori* Cag T4SS (3, 10, 12, 61).

In previous studies, we have utilized mutagenesis approaches to gain insight into the contributions of individual OMCC proteins to the organization and activity of the Cag T4SS (9, 61, 62); however, there are many unresolved questions, including the roles of species-specific protein adaptations in Cag T4SS architecture and activity. In the current study, we focus on CagX, a VirB9 homolog. VirB9 proteins are important components of bacterial T4SSs (42, 43) (Fig. S1) that play key roles in substrate selection and substrate secretion (63). Early studies showed that VirB9 is an integral component of the OMCC in prototype T4SSs. In *Agrobacterium tumefaciens,* the N-terminal region of VirB9 has a role in substrate selection, while the C-terminal region has a role in substrate transfer (63). *H. pylori* CagX is of particular interest because it is larger in size than VirB9 proteins of other bacterial T4SSs and contains regions with weak sequence relatedness (Fig. S1B), as well as a 130-amino-acid species-specific insertion, designated as the periplasmic insertion (PI) sequence (Fig. 1B). Most of the CagX structure (over 70%) has been resolved by single particle cryo-EM (10, 12), but the structure of the PI domain was not visualized in a previously determined Cag T4SS OMCC cryo-EM 3D density map (10, 12). These observations suggest that CagX contains domains that are conserved across VirB9 proteins, as well as flexible or unstructured regions that are unique to *H. pylori*.

In this study, we set out to understand the contributions of conserved and *H. pylori-*specific domains of CagX to the overall organization and activity of the Cag T4SS. By generating *H. pylori* strains in which VirB9-conserved domains and an *H. pylori*-specific domain are individually deleted, we define the contributions of individual CagX domains to the activity and structural organization of the Cag T4SS OMCC.

## RESULTS

### Requirement of conserved CagX domains for Cag T4SS activity

**Fig 2:**
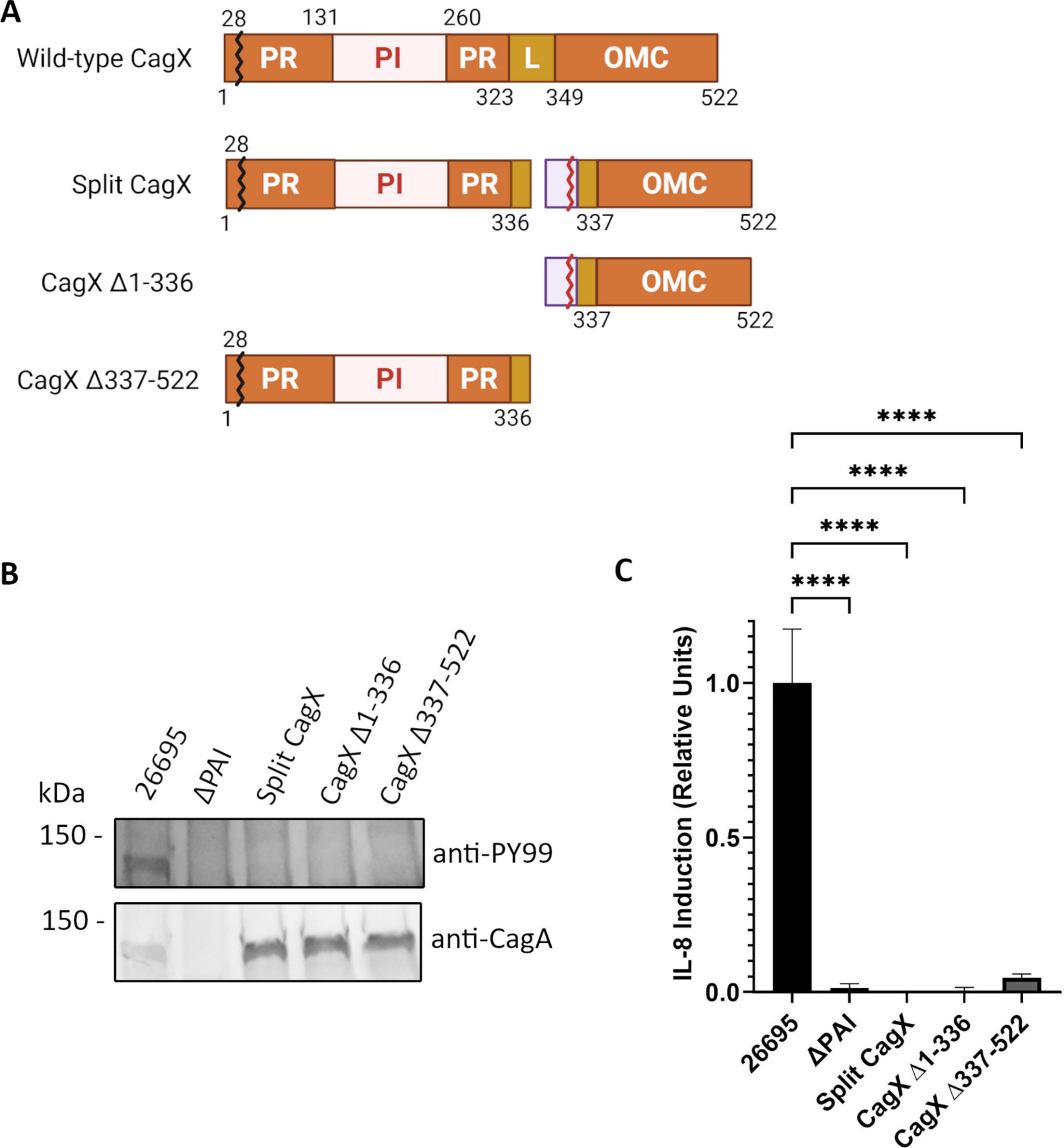
Conserved domains of CagX are required for Cag T4SS activity. (**A**) Schematic of CagX proteins encoded by mutants generated in this study. CagX Δ1–336 (CDO1): deletion of the PR domain of CagX. CagX Δ337–522 (CDO2): deletion of the OMC domain of CagX. Split CagX (CDO3): disrupting the CagX linker region to express the PR and OMC domains as two independent fragments. This numbering system includes the CagX signal sequence (residues 1–28, based on SignalP 6.0 prediction; cleavage site indicated by black zig-zag lines). The signal sequence of the VacA toxin (residues 1–33; cleavage site indicated by red zig-zag lines) was inserted upstream of the sequences encoding the CagX OMC domain to ensure protein secretion across the inner membrane. DNA sequences encoding modified forms of CagX were introduced into an *H. pylori* strain SCT72 that also produces HA-CagF (CagF with a hemagglutinin [HA] tag). Additional details are shown in Fig. S2. PR, periplasmic ring. PI, periplasmic insertion domain. L, linker. OMC, outer membrane cap. (**B**) The generated CagX mutant strains CagX Δ1–336 (CDO1), CagX Δ337–522 (CDO2), and Split CagX (CDO3), as well as the wild-type strain (26695) and a strain lacking the *cag* pathogenicity island (∆PAI), were co-cultured with AGS cells. CagA translocation was assessed by probing for phosphorylated CagA (anti-PY99) and CagA (CagA antiserum). (**C**) *H. pylori* strains were co-cultured with AGS cells, and Cag T4SS activity was assessed by using an IL-8 ELISA. Statistical significance was determined by a one-way ANOVA with Dunnett’s test for multiple comparisons. ****P < 0.0001.

When compared with VirB9 homologs from other bacterial T4SSs, CagX shares three structurally conserved features—an N-terminal β-sheet region that contributes to the PR, a C-terminal β-sandwich fold found in the OMC, and a linker region that bridges the OMC and PR (Fig. 1B; Fig. S1B) (12, 64). In addition, CagX contains a 130-amino-acid species-specific region (designated as the PI domain), which is not present in VirB9 proteins from other bacterial species (Fig. 1B; Fig. S1B). Previous cryo-EM studies delineated the structure of CagX within the OMC and PR, but the linker region was incompletely resolved, and the PI domain was not visualized (12). To investigate the contributions of the conserved regions of CagX found in the OMC and PR to Cag T4SS structure and function, we generated *H. pylori* mutant strains in which the portions of CagX mapped to either the PR (CagX ∆1–336; strain CDO1) or OMC (CagX ∆337–522; strain CDO2) were deleted (Fig. 2A and Table 1). Additionally, we disrupted the linker region so that the OMC and PR domains could be produced in the same strain as two separate proteins (Split CagX, strain CDO3; Fig. 2A; Fig. S2; Table 1). In each case, the mutant strains were derived from strain SCT72, a modified 26695 strain that produces an HA-epitope-tagged CagF protein. Western blotting confirmed that the mutant strains produced truncated forms of CagX of the expected sizes (Fig. S3).

**TABLE 1:** *H. pylori* strains used in this study

Strain name	Description	Genotype	Reference
26695	Wild-type: Intact *cag* PAI	Wild-type strain 26695	(65, 66)
ΔPAI	∆PAI: Deletion of *cag* PAI	26695 Δ(*cagA-cag1)* (Chl^R^)	(66)
HA-CagF 26695	Wild-type CagX: Expresses HA-CagF in the *ureAB* chromosomal locus	26695 Δ*cagF* Δ*rdxA* (Mtz^R^) *ureA::*HA-*cagF* (Chl^R^)	(9)
SCT72	Introduced HA-CagF and gentamicin resistance cassette into HP0630 and HP0631 intergenic locus	26695 HA-*cagF* and *aac (3)-IV* inserted into *hydA-mdaB* intergenic region (Gent^R^)	This study
HA-CagF Δ*cagX*	Δ*cagX:* Introduced HA-CagF into the *ureAB* chromosomal locus in an unmarked ∆*cagX* mutant strain	26695 Δ*rpsL* (Str^R^) Δ*cagX ureA::*HA-*cagF* (Chl^R^)	(9, 67)
CDO1	CagX ∆1–336: Deletion of the PR domain of CagX (amino acids 1–336) in strain SCT72 bearing HA-CagF	SCT72 *cagX*(Δ1–336) (Chl^R^), *PcagV-Y*::*vacA* (1–33):: FLAG-*cagX*(337–522)	This study
CDO2	CagX ∆337–522: Deletion of the OMC domain of CagX (amino acids 337–522) in strain SCT72 bearing HA-CagF	SCT72 *cagX(1–336)-FLAG,* *cagX*(Δ337–522) (Chl^R^) P*cagV-Y*::*cagY*	This study
CDO3	Split CagX: Expressed CagX OMC and PR domains as two independent fragments in strain SCT72 bearing HA-CagF	SCT72 *cagX*(1–336)-FLAG, (Chl^R^), *PcagV-Y*::*vacA* (1–33):: *cagX*(337–522)	This study
CDO4	CagX ∆131–260: Deletion of the *H. pylori-*specific CagX periplasmic insertion (PI; amino acids 131–260) in the strain bearing HA-CagFΔ*cagX*	HA-CagF Δ*cagX* P*cagV-Y*::*cagX*(1–130)::FLAG::*cagX*(261–522) replaces *rdxA*	This study

Next, we tested the consequence of these mutations on Cag T4SS activity in *H. pylori*-AGS cell co-culture assays. When *H. pylori* strains expressing CagX ∆1–336, CagX ∆337–522, or Split CagX were co-cultured with AGS gastric epithelial cells, all three mutant strains lacked the ability to translocate CagA (Fig. 2B) and induce IL-8 secretion (Fig. 2C). These results indicate that an intact CagX protein, containing both the PR and OMC domains, is required for Cag T4SS activity.

### Role of conserved CagX domains in Cag T4SS OMCC formation

**Fig 3:**
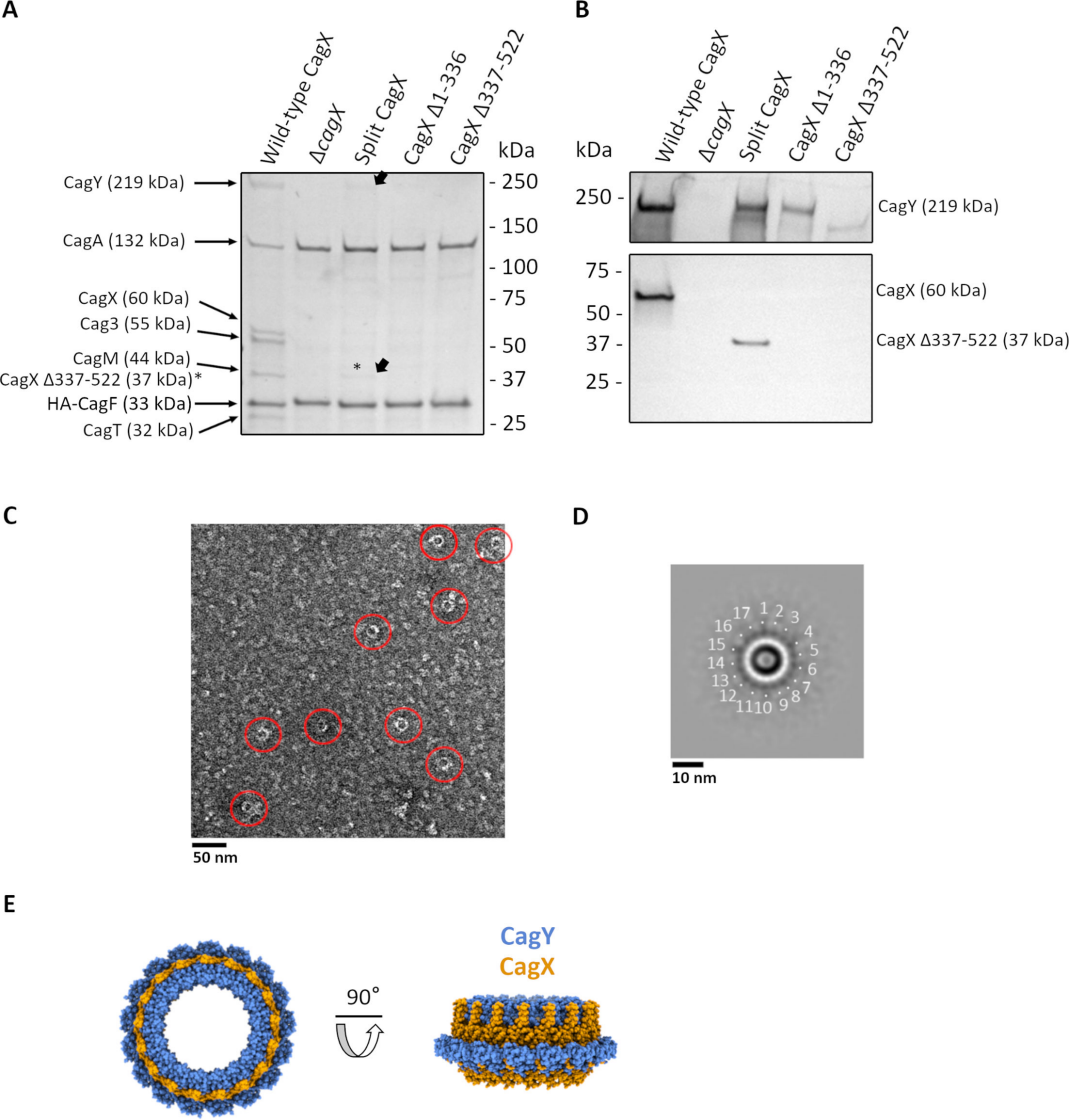
The Cag T4SS PR (consisting of 17 copies of CagX and CagY) can assemble when CagX is disrupted within the linker region. Preparations of Cag T4SS OMCCs immunopurified from strains producing wild-type CagX (HA-CagF 26695), *ΔcagX* (HA-CagF Δ*cagX*), CagX Δ1-336 (CDO1), CagX Δ337-522 (CDO2), or Split CagX (CDO3) were assessed as described in methods. (**A**) OMCC samples were resolved by SDS-PAGE and stained with Coomassie blue. Bands corresponding to CagY (219 kDa) and the CagX PR domain (CagX Δ337–522; 37 kDa) were weakly detected in the preparation from the Split CagX mutant, as indicated by the black arrows. *, marks the position of CagX Δ337–522 in the Coomassie-stained gel. (**B**) Immunoblot using antibodies to CagY (top) and CagX (bottom) confirmed copurification of CagX and CagY from the Split CagX mutant. See corresponding mass spectrometry analyses of immunopurified samples shown in Table 2; Fig. S4. A smaller size CagY protein was produced by CagX ∆337–522 mutants generated in two independent experiments; mass spectrometry analysis indicated a truncation in the N-terminal region of CagY. (**C**) Representative negative stain electron microscopy images of complexes isolated from the Split CagX mutant strain revealed complexes that are ~19 nm in diameter. (**D**) Representative 2D classification of negative-stained particles from the Split CagX mutant revealed 17-fold symmetry, which is characteristic of the PR. (**E**) Structures of CagX and CagY within the PR subassembly of a wild-type Cag T4SS OMCC, based on a previous cryo-EM analysis (PDB: 6X6J). Views are shown from outer to inner membrane and rotated 90˚ to show the side view.

To determine the structural contributions of the conserved domains of CagX to the organization of the Cag T4SS OMCC, we performed immunoprecipitation experiments on HA-CagF-encoding strains that produce wild-type CagX (HA-CagF 26695), CagX ∆1–336 (CDO1), CagX ∆337–522 (CDO2), Split CagX (CDO3), or no CagX (HA-CagF ∆*cagX*) (Table 1), using antibodies to the HA-epitope tag on CagF (9). When the immunopurified preparations were resolved on SDS-PAGE gels and stained with Coomassie blue, multiple bands corresponding to Cag T4SS OMCC components could be detected in the preparation from the wild-type CagX-producing strain but not from the ∆*cagX* mutant (HA-CagF ∆*cagX*), as expected (Fig. 3A). CagY and a modified CagX band, corresponding to the expected 37 kDa size of the PR domain (CagX ∆337–522), were weakly detectable in the preparation from the Split CagX *H. pylori* mutant strain, but not in preparations from CagX ∆1–336 or CagX ∆337–522 mutants (Fig. 3A). Immunoblotting analysis confirmed that CagX and CagY were present in the preparation from the Split CagX mutant (Fig. 3B). We also analyzed the immunopurified samples using tandem mass spectrometry (Table 2). As expected, we detected spectral counts for CagY, CagX, CagT, CagM, and Cag3, as well as CagW, CagH, CagI, and CagL in preparations of the wild-type OMCC. Consistent with the Coomassie stain and immunoblotting results (Fig. 3A and B), OMCCs from the wild-type strain and preparations from the Split CagX mutant contained higher spectral counts of CagX than preparations from the CagX ∆1–336 or CagX ∆337–522 mutants (Table 2). Tandem mass spectrometry analysis showed that both CagX and CagY were present in OMCCs purified from the Split CagX mutant (Table 2). An analysis of mass spectrometry peptide sequence coverage showed that peptides corresponding to both the OMC and PR regions of CagY and CagX were detected in preparations from the Split CagX mutant (Fig. S4). In contrast, other OMC structural components (CagM, CagT, and Cag3) and minor T4SS components (CagW, CagH, CagI, and CagL) were not detected or minimally detected in the mass spectrometry analysis of OMCCs from the Split CagX mutant (Table 2).

**TABLE 2:** LC-MS/MS analysis of proteins immunoprecipitated from strains producing the indicated CagX proteins

Identified Proteins*^a^*	Wild-type CagX*^b^*	∆*cagX^b^*	CagX ∆1–336*^b^*	CagX ∆337–522*^b^*	Split CagX*^b^*	CagX ∆131–260*^b^*
CagF	378	313	544	519	450	312
CagA	3217	4282	5774	6580	6312	5340
CagX	1203	0	8	77	382	1172
CagY	2423	9	520	251	1503	4154
CagM	974	2	1	0	0	1203
CagT	1167	0	0	0	1	310
Cag3	1394	0	0	0	0	391
CagW	29	0	2	0	7	0
CagH	19	0	0	0	1	0
CagI	32	1	2	1	1	2
CagL	16	0	0	0	0	0
CagE	36	5	14	4	26	41
Cag5	27	5	24	7	31	18
CagV	23	6	14	14	21	22
CagZ	2	2	8	0	12	0
CagN	14	0	1	0	1	7
Cag1	4	4	1	3	5	2
CagS	2	2	1	0	5	6
Cagα	5	0	20	13	20	5
No. of Cag Spectra	10,587	4,318	6,390	6,950	8,328	12,673
% Cag Spectra	52%	45%	42%	57%	48%	54%
Total Spectral Counts	20,212	9,496	15,331	12,087	17,403	23,384

^*a*^ Cag proteins were detected by tandem mass spectrometry. Numbers represent spectral counts.

^*b*^ Proteins were isolated by immunoprecipitation of HA-CagF from strains producing the indicated CagX proteins, as described in the Methods.

We next visualized the immunopurified preparations from the Split CagX strain using negative stain EM and 2D classification. This analysis showed that purified complexes from the Split CagX mutant did not look like intact OMCCs, but instead consisted of circular rings (~19 nm in diameter) with 17 visible repeating units, which structurally resembled the PR in the single-particle cryo-EM structure of the Cag T4SS (Fig. 3C through E; Fig. S5). Periplasmic rings with similar morphology were detected in our previous analysis of ∆*cagT* and ∆*cagM* mutants (9, 61). These results indicate that an intact CagX protein (containing both the PR and OMC domains) is required to assemble a complete OMCC and indicate that the PR of the Cag T4SS, consisting of 17 copies of CagX and CagY, can stably assemble in the absence of a fully structured OMC subassembly. In addition, these results provide evidence that an intact CagX protein is required for the association of CagW, CagH, CagI, and CagL with the OMCC.

### Analysis of the *H. pylori*-specific CagX periplasmic insertion (PI) domain

**Fig 4:**
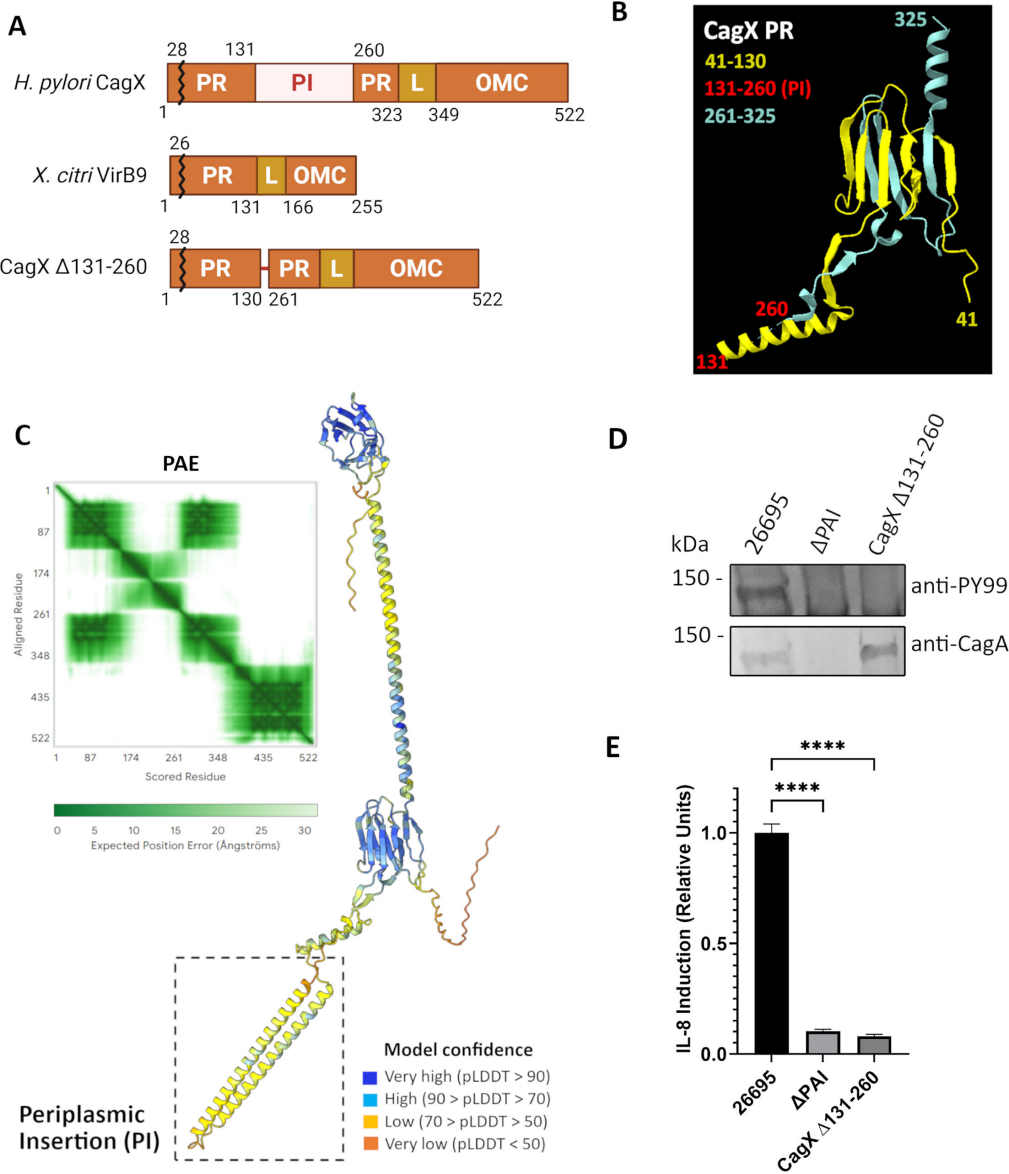
The periplasmic insertion (PI) domain of CagX is unique to *H. pylori* and required for Cag T4SS activity. (**A**) An *H. pylori* species-specific PI domain (residues 131–260) comprises about 25% of CagX. The schematic compares CagX to *X. citri* VirB9. Signal sequences are illustrated (CagX residues 1–28, *X. citri* VirB9 residues 1–26; based on SignalP 6.0 prediction). The third schematic illustrates the engineered mutant CagX Δ131–260 (CDO4): deletion of the 130 amino-acid *H. pylori* species-specific PI domain of CagX. (**B**) The CagX PI domain could not be modeled in the CagX structure from previous cryo-EM studies of the Cag T4SS OMCC (PDB: 6X6J). (**C**) AlphaFold 3 (68) prediction of one copy of CagX, including the unresolved PI domain, colored by pLDDT – predicted Local Distance Difference Test. PAE, Predicted Aligned Error; predicted template modeling score (pTM = 0.38). (**D**) *H. pylori* wild-type (26695), a strain lacking the *cag* pathogenicity island (∆PAI), and CagX Δ131–260 (CDO4) were co-cultured with AGS cells, and CagA translocation was assessed by probing for phosphorylated CagA (anti-PY99) and CagA (CagA antiserum). (**E**) Cag T4SS activity was also assessed by using an IL-8 ELISA. Statistical significance was determined by a one-way ANOVA with Dunnett’s test for multiple comparisons. ****P < 0.0001.

Amino acid sequence alignment of CagX with VirB9 proteins from minimized T4SSs of *A. tumefaciens* and *X. citri* reveals a 130-amino-acid region in CagX that lacks homology to prototypical VirB9 proteins (Fig. 4A; Fig. S6A). This region, designated as the *H. pylori-*specific CagX periplasmic insertion (PI), is located between two regions of CagX that localize to the PR domain (12). The CagX PI domain corresponds to about 25% of the structure of CagX and has not been resolved by single particle cryo-EM (Fig. 4B). There are multiple possible reasons to explain why the structure of this region has not yet been resolved. For example, the CagX PI domain might be localized to regions of the OMCC that are either flexible or unstable after purification from the bacterial membranes. The PI sequence is highly conserved among *H. pylori* strains (Fig. S6B), suggesting that it might be important for function. However, the role of this domain in Cag T4SS structure and function has not been investigated.

In the cryo-EM 3D structure, there was no detectable density for the CagX PI residues; hence, we used AlphaFold 3 (68) to predict the structure of this region in the context of full-length CagX (Fig. 4C). AlphaFold 3 predicts that the PI domain folds into two alpha helices localized near the PR region of CagX (Fig. 4C).

### Contributions of the CagX PI domain to Cag T4SS activity and OMCC morphology

To investigate the contribution of the *H. pylori*-specific CagX PI domain to the overall organization and activity of the Cag T4SS, we generated an *H. pylori* mutant in which the CagX PI domain (residues 131–260) is deleted (CagX ∆131–260, strain CDO4; Fig. 4A; Fig. S2; Table 1). Immunoblotting with antiserum to CagX demonstrated production of the truncated CagX protein of the expected size (Fig. S7). When co-cultured with AGS gastric epithelial cells, the *H. pylori* CagX ∆131–260 mutant could neither translocate CagA into gastric epithelial cells (Fig. 4D) nor induce IL-8 secretion (Fig. 4E), indicating that the PI domain is required for Cag T4SS activity.

**Fig 5:**
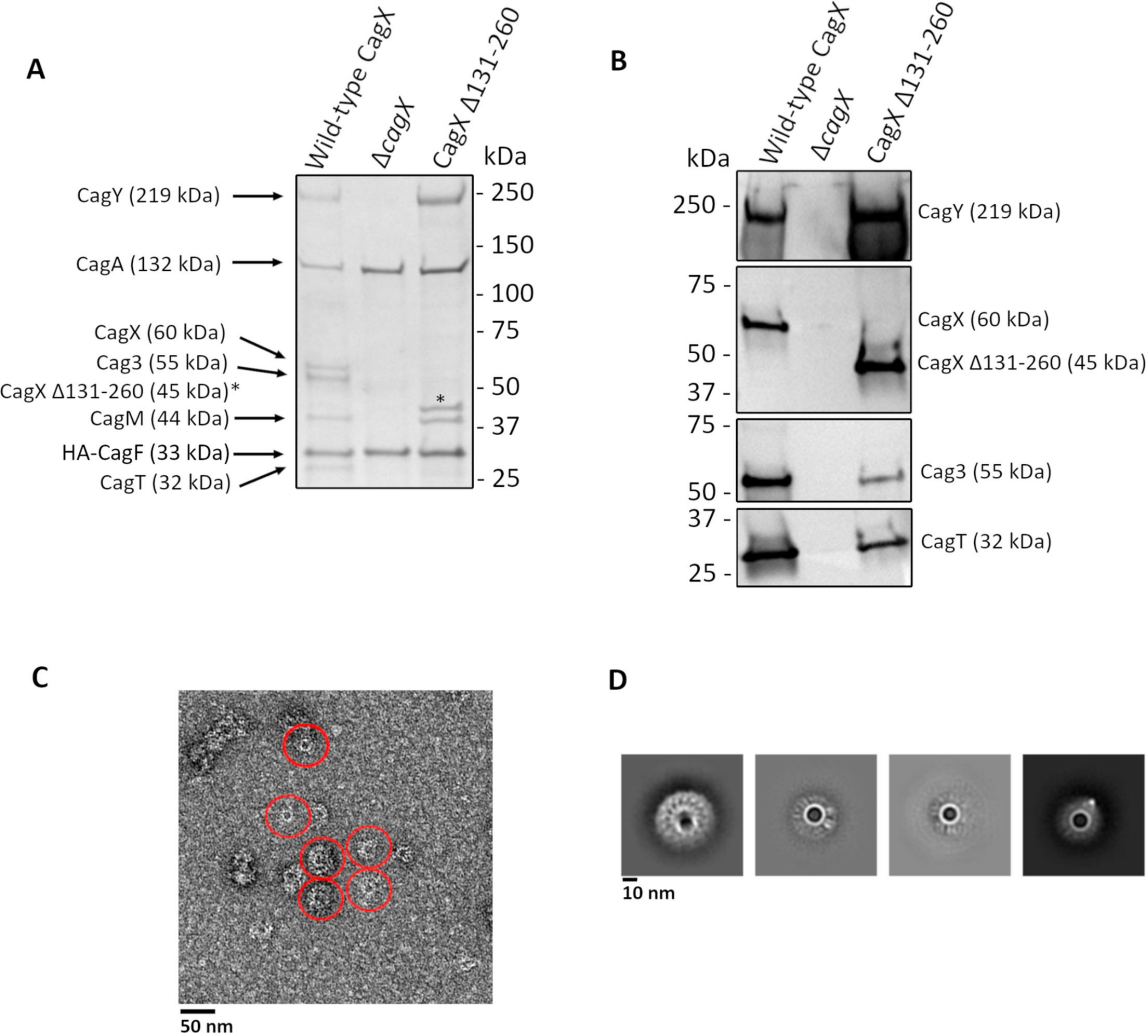
Partially assembled Cag T4SS OMCCs are isolated from an *H. pylori* mutant lacking the CagX PI domain (CagX Δ131–260; CDO4). (**A**) Preparations of Cag T4SS OMCCs were immunopurified from strains producing the indicated CagX proteins. Coomassie-stained SDS-PAGE gel shows multiple bands corresponding to Cag T4SS OMCC components from the CagX Δ131–260 mutant, with differences in protein bands corresponding to CagX, CagT, and Cag3 when compared to wild-type complexes. *, marks the position of CagX ∆131–260 in the Coomassie-stained gel. (**B**) Immunoblots of OMCC preparations from the wild-type CagX strain and the CagX Δ131–260 mutant show the expected differences in CagX sizes (wild-type CagX, 60 kDa; CagX Δ131–260, 45 kDa). (**C**) Representative electron micrograph of negative-stained OMCCs purified from the CagX Δ131–260 mutant. (**D**) Representative 2D class averages of negative-stained CagX Δ131–260 OMCCs showing *en face* views.

To determine if the CagX PI domain is required for OMCC formation, we performed immunoprecipitation experiments with the CagX ∆131-260 mutant, which also produces HA-epitope-tagged CagF (strain CDO4). The resulting preparations were analyzed by SDS-PAGE and staining using Coomassie blue (Fig. 5A). Multiple bands, corresponding to components of the OMCC, copurified with CagA and HA-CagF from the wild-type CagX-producing control strain and from the CagX ∆131–260 mutant, although to different extents. As expected, a 60 kDa band corresponding to wild-type CagX was detected in complexes purified from the wild-type CagX-producing strain, and a 45 kDa band (corresponding to truncated CagX) was detected in complexes purified from the CagX ∆131–260 mutant. Bands corresponding to the expected sizes of CagY, CagM, HA-CagF, and CagA were also detected by Coomassie stain in preparations from both the wild-type CagX-producing strain and the CagX ∆131–260 mutant. However, OMCC bands corresponding to Cag3 and CagT were not detected by Coomassie stain in preparations from the CagX ∆131–260 mutant.

Immunoblots using antisera to Cag3 and CagT revealed that the respective bands for Cag3 and CagT could be detected at the expected sizes in preparations of the CagX ∆131–260 OMCCs but were present in lower relative abundance compared to preparations of wild-type OMCCs (Fig. 5B). Tandem mass spectrometry analysis revealed that preparations of CagX ∆131–260 OMCCs contained peptides corresponding to the CagX PR domain (41–130; 261–336) and OMC domain (337–522), but not peptides corresponding to the CagX PI domain (residues 131–260), as expected (Fig. S4). Consistent with results of the Coomassie-stained gel and immunoblotting experiments, tandem mass spectrometry analysis of OMCCs purified from the CagX ∆131–260 mutant revealed reduced spectral counts for CagT and Cag3 relative to preparations from the wild-type strain (Table 2). While CagW, CagH, CagI, and CagL were detected in preparations from the wild-type strain, minimal spectral counts corresponding to these four proteins were detected in preparations from the CagX ∆131–260 mutant.

**Fig 6:**
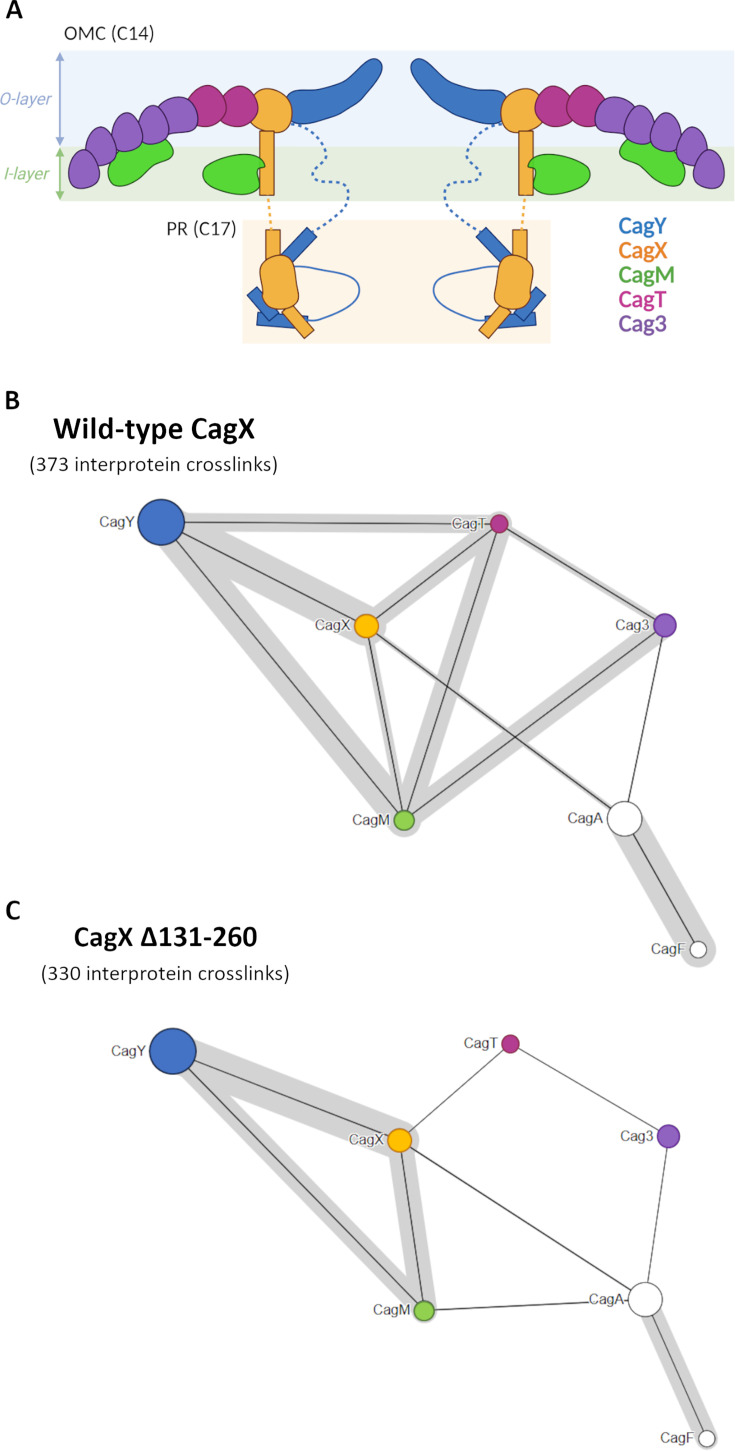
Protein-protein interactions maintained or altered in the absence of the species-specific CagX PI domain. (**A**) The schematic, adapted from a previous study (12), illustrates locations of individual proteins within the wild-type OMCC. The inner (I)-layer of the OMC is formed by interactions between the OMC domains of CagX and CagY with CagM, while the outer (O)-layer involves interactions of OMC domains of CagX and CagY with CagT and Cag3. (**B and C**) OMCCs were purified from wild-type CagX (HA-CagF 26695) and CagX Δ131–260 (CDO4) mutant strains, then treated with the amine-reactive crosslinker BS3 and analyzed by LC-MS/MS. Sites of interprotein crosslinks between OMCC components were identified using the pLink 3 program (69). Network maps, depicted using xiNET cross-link viewer (70), illustrate the identified BS3 interprotein crosslinks between proteins in (**B**) wild-type OMCCs (373 total crosslinks) and (**C**) CagX Δ131-260 OMCCs (330 total crosslinks). See Tables S2 and S3 for further details. The thickness of gray connections is proportional to the total number of interprotein crosslinks detected between two proteins. The diameter of each circle corresponds to the molecular weight of each protein.

Negative stain electron microscopy analysis of purified complexes from the CagX ∆131–260 mutant revealed an assortment of particles, including some complexes that appear to have a nearly intact structure, similar to that of wild-type OMCCs, as well as ring-like complexes that were smaller than wild-type OMCCs (9) (Fig. 5C and D; Fig. S8). The rings were about 19 nm in diameter and resembled complexes formed by Δ*cagT* and Δ*cagM* mutants (61), which correspond to the 17-fold-symmetric PR portion of the OMCC. Some of the complexes appeared to contain not only the PR ring but also peripheral finger-like projections and/or partially assembled OMCs. We interpret the projections to likely correspond to the inner-layer (I-layer) of the OMC subassembly, which consists of CagM (Fig. 6A) (12). These results suggest that the O-layer of the OMC subassembly is destabilized in the absence of the CagX PI domain, resulting in heterogeneously organized OMCs compared to the assembled PR.

In summary, the CagX ∆131–260 mutant retained the ability to form OMCCs containing five OMCC components (CagX, CagY, CagM, CagT, and Cag3; Table 2), and EM analysis indicated that the structures had an intact PR (Fig. 5). Conversely, OMCCs formed by the CagX ∆131–260 mutant differed from wild-type OMCCs in several ways, including reduced content of CagT and Cag3; absence of detectable CagW, CagH, Cagl, and CagL; and a destabilized OMC portion of the OMCC, based on EM analysis.

### Protein-protein interactions involving CagX

The CagX PI domain was not visualized in previous cryo-EM studies, which suggests that this is a flexible region in preparations of purified OMCCs. Since the CagX ∆131–260 mutant (CDO4) retained the ability to assemble partially intact OMCCs, we hypothesized that the CagX ∆131–260 protein (lacking the PI domain) retains the ability to physically interact with other OMCC proteins in both the OMC and PR. To test this hypothesis, we undertook crosslinking mass spectrometry experiments designed to analyze the spatial relationships of wild-type CagX with other proteins in the OMCC, compared to those involving CagX ∆131–260.

Purified wild-type OMCCs and CagX ∆131–260 mutant OMCCs were treated with two different concentrations (0.1 and 0.25 mM) of the water-soluble crosslinker bis-sulfosuccinimidyl suberate (BS3), which crosslinks proteins at primary amines (e.g., lysines). Coomassie stain analysis revealed that bands corresponding to individual OMCC components were reduced in intensity in BS3-treated samples compared to control samples without BS3 treatment (Fig. S9). BS3-treated protein complexes were analyzed by tandem mass spectrometry (Table S1), and the resulting data were analyzed using pLink 3 software (69) to identify sites of interprotein crosslinks between OMCC proteins.

**Fig 7:**
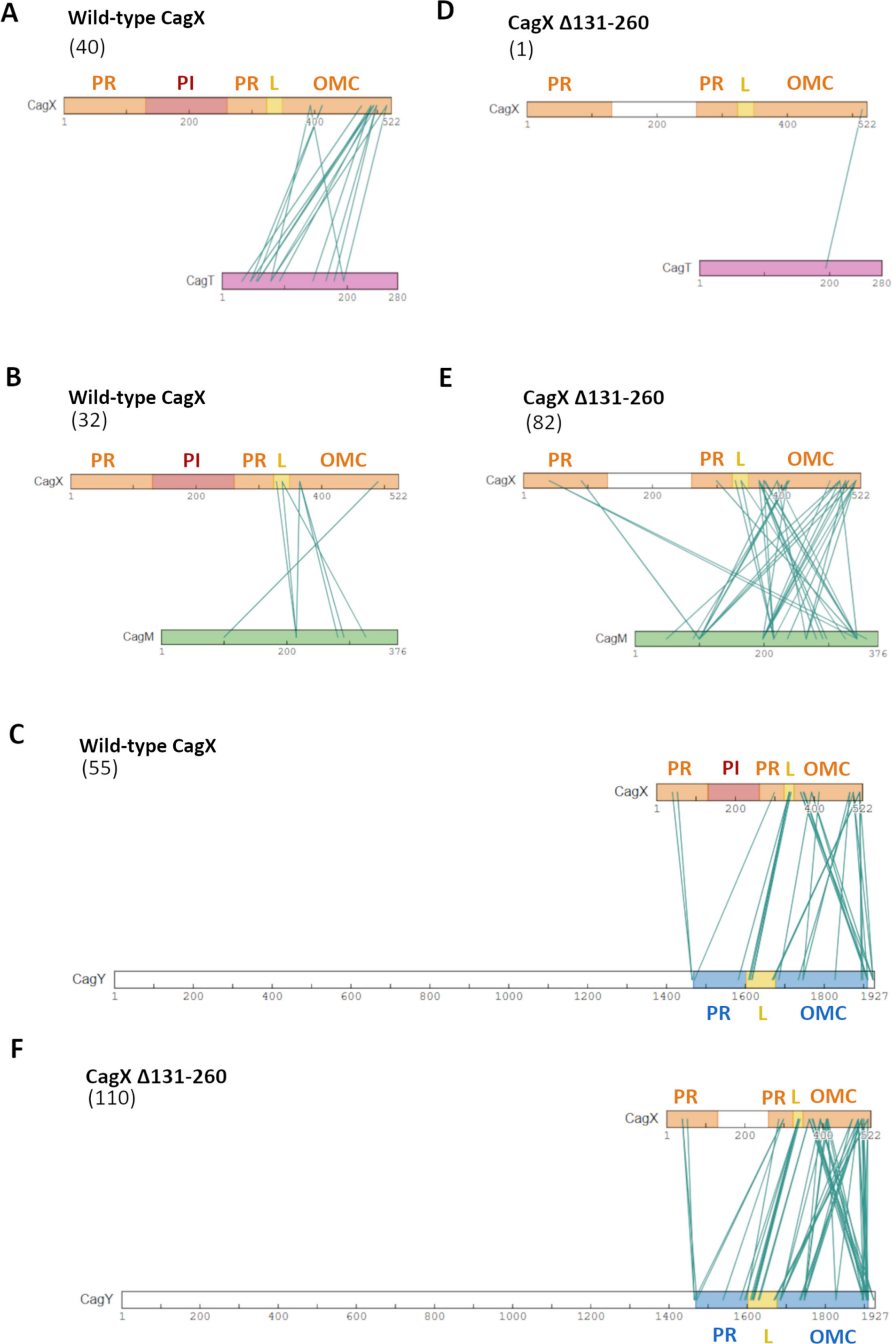
Schematics depicting BS3 lysine-lysine interprotein crosslinks in preparations of OMCCs from the (**A–C**) wild-type CagX strain (HA-CagF 26695) or (**D–F**) the CagX Δ131–260 mutant (lacking the PI domain; CDO4). Lines indicate the sites of crosslinks. Numbers in parentheses represent the total number of interprotein crosslinks identified between the indicated protein pairs: (**A and D**) CagX-CagT, (**B and E**) CagX-CagM, and (**C and F**) CagX-CagY. See Tables S2 and S3 for further details. PI, periplasmic insertion (red). L, linker region (yellow). OMC, outer membrane cap (CagX – orange; CagY – blue). PR, periplasmic ring (CagX – orange; CagY – blue).

In a representative crosslinking mass spectrometry analysis (pooling results using two different concentrations of the BS3 crosslinker for each preparation), we detected 373 total (164 unique) interprotein crosslinks in the BS3-treated wild-type OMCC sample (Fig. 6B; Table S2). The total number of interprotein crosslinks includes repetitive detection of individual crosslinked lysine-lysine pairs. No interprotein crosslinks were detected in samples from a parallel control experiment without BS3 treatment. We detected eight sets of interprotein crosslinks among OMCC components (CagX-CagY, CagX-CagM, CagX-CagT, CagY-CagT, CagY-CagM, CagM-CagT, CagM-Cag3, and CagT-Cag3) (Fig. 6B; Table S2). These interactions are consistent with the known positions of these proteins within the OMCC, based on models built from the cryo-EM analysis (12). Conversely, interprotein crosslinks between CagX and Cag3 were not detected, consistent with non-contiguous localization of these proteins in the OMCC (Fig. 6B). The detection of interprotein crosslinks between HA-CagF (the epitope-tagged bait protein used for the immunopurification experiments) and CagA is consistent with the known interactions of CagF with CagA (Fig. 6B) (9, 51). Interprotein crosslinks involving CagW, CagH, CagI, or CagL were not detected, perhaps due to the low abundance of these proteins. In the current study, we focused on CagX interactions (CagX-CagY, CagX-CagT, and CagX-CagM) (Fig. 6 and 7). In an analysis of wild-type OMCCs, we detected interprotein crosslinks between the OMC domain of CagX with CagT (Fig. 7A), as well as crosslinks between the CagX OMC and linker regions with CagM (Fig. 7B), consistent with the known localization of CagT to the O-layer of the OMC and localization of CagM to the I-layer of the OMC (Fig. 6A) (12). Notably, we did not detect interprotein crosslinks between the CagX PI domain and other components of the Cag T4SS OMCC (Fig. 7A through C; Table S2).

We detected 330 total (159 unique) interprotein crosslinks in the analysis of a BS3-treated OMCC preparation from the CagX PI deletion mutant (Fig. 6C; Table S3). We detected interprotein crosslinks between CagX-CagY and CagX-CagM in OMCCs from the CagX Δ131–260 mutant (Fig. 6C), similar to results with the wild-type OMCCs (Fig. 6B). Specifically, we detected eight identical CagX-CagM interprotein crosslinks (Table 3) and 24 identical CagX-CagY interprotein crosslinks (Table 4) in both wild-type and CagX ∆131–260 mutant OMCCs. These results indicate that the CagX PI domain is not required for CagX interactions with CagY or CagM (Fig. 6C, 7E and 7F), suggesting that the modified CagX protein (lacking the PI domain) retains a folded structure that allows these protein-protein interactions to occur.

**TABLE 3:** CagX-CagM BS3 interprotein crosslinks common to both wild-type and CagX ∆131–260 complexes^*a*^

CagX lysine position	CagX domain	CagM lysine position	Wild-type CagX^*b*^	CagX ∆131–260^*b*^
328	Linker	215	4	4
337	Linker	215	5	5
337	Linker	326	2	1
365	OMC	291	1	3
365	OMC	215	2	3
365	OMC	291	16	3
365	OMC	281	2	2
490	OMC	100	1	3
Total BS3 interprotein crosslinks			33	24

^*a*^ OMCCs were immunopurified using HA-CagF from strains producing the indicated CagX proteins, prior to treatment with the crosslinker BS3 at 0.1 mM or 0.25 mM concentrations. Crosslinked OMCCs were analyzed by tandem mass spectrometry and processed through pLink 3 (69) to identify sites of interprotein crosslinks.

^*b*^ Numbers represent the total number of corresponding CagX-CagM interprotein BS3 crosslinks detected in each immunopurified preparation.

**TABLE 4:** CagX-CagY BS3 interprotein crosslinks common to both wild-type and CagX ∆131–260 complexes^*a*^

CagX lysine position	CagX domain	CagY lysine position	CagY domain	Wild- type CagX^*b*^	CagX ∆131–260^*b*^
40	PR	1,465	N-term	3	4
53	PR	1,465	N-term	4	4
299	PR	1,471	PR	2	3
337	Linker	1,582	PR	2	1
337	Linker	1,608	Linker	2	2
337	Linker	1,612	Linker	2	2
337	Linker	1,617	Linker	2	3
341	Linker	1,617	Linker	2	2
365	OMC	1,910	OMC	1	2
365	OMC	1,924	OMC	2	2
372	OMC	1,910	OMC	2	1
374	OMC	1,910	OMC	2	2
393	OMC	1,685	OMC	4	2
393	OMC	1,910	OMC	1	2
393	OMC	1,924	OMC	4	4
412	OMC	1,746	OMC	2	2
490	OMC	1,746	OMC	2	2
490	OMC	1,827	OMC	1	1
499	OMC	1,734	OMC	2	3
499	OMC	1,910	OMC	2	2
515	OMC	1,668	Linker	1	3
515	OMC	1,671	Linker	2	2
515	OMC	1,671	Linker	2	4
515	OMC	1,894	OMC	2	4
Total BS3 interprotein crosslinks				51	59

^*a*^ OMCCs were immunopurified using HA-CagF from strains producing the indicated CagX proteins, prior to treatment with the crosslinker BS3 at 0.1 mM or 0.25 mM concentrations. Crosslinked complexes were analyzed by LC-MS/MS and processed through pLink 3 (69) to identify sites of interprotein crosslinks.

^*b*^ Numbers represent the total number of corresponding CagX-CagY interprotein BS3 crosslinks detected in each immunopurified preparation.

Notably, when the CagX PI domain is deleted, the number of interprotein CagX-CagT crosslinks is markedly reduced (Fig. 7D), while the numbers of CagX-CagM (Fig. 7E) and CagX-CagY (Fig. 7F) crosslinks are increased. Decreased CagX-CagT interactions likely contribute to destabilization of the OMC, consistent with a loss of CagM-Cag3 and CagT-Cag3 interactions (Fig. 6). In addition, the reduced abundance of Cag3 and CagT in the CagX ∆131–260 mutant OMCCs probably increases accessibility of the crosslinker for CagX and CagM. The observed disruptions of CagT and Cag3 interactions within the OMC in the absence of the CagX PI domain mirror the results of the biochemical and EM analyses shown in Fig. 5 and Table 2. Collectively, these results indicate that the CagX PI is not required for CagX interactions with CagY or CagM but indicate that the CagX PI is required for stable assembly of the OMC, perhaps by bridging and/or stabilizing connections among components of the O-layer of the Cag T4SS OMC (including CagT and Cag3).

**Fig 8:**
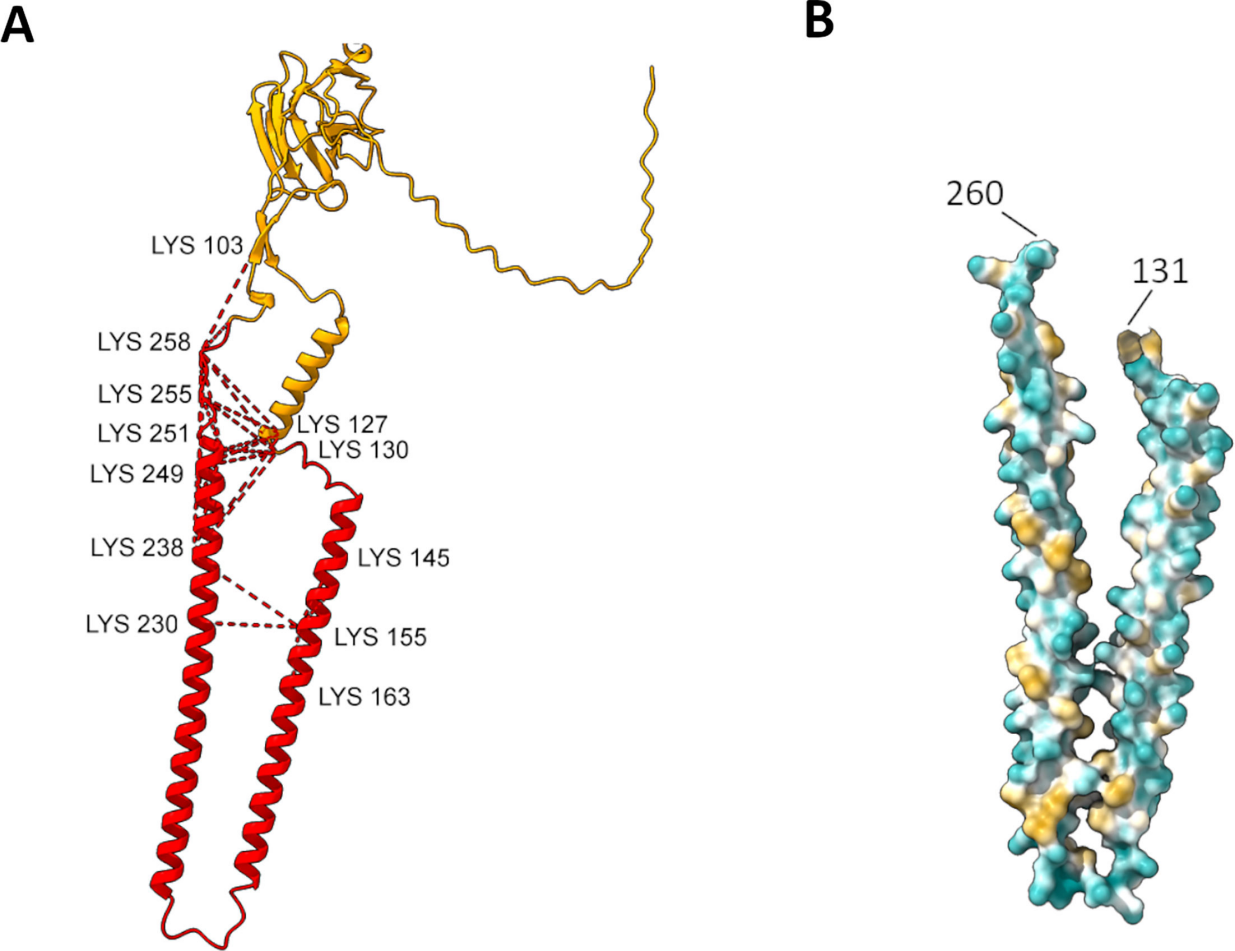
Analysis of the AlphaFold 3-predicted structure of the CagX PI domain. (**A**) Representative BS3 intraprotein crosslinks involving the CagX PI domain (red). OMCCs were purified from a strain producing wild-type CagX (HA-CagF 26695) and treated with BS3 prior to analysis by tandem mass spectrometry. Lysine-lysine intraprotein crosslinks were detected by analysis using the pLink 3 program (69) and mapped into the AlphaFold 3 (68) prediction of CagX. A total of 27 intraprotein crosslinks within 30 Å (71), corresponding to dashed lines, are shown. See further details in Table S4. (**B**) Hydrophobicity analysis of the CagX PI region (residues 131–260) predicted by AlphaFold 3 reveals a relatively hydrophobic α-helical strand (yellow) and a relatively hydrophilic α-helical strand.

AlphaFold 3 modeling suggests that the CagX PI folds into two α-helices (Fig. 4C and 8). To test the validity of this model, we examined the crosslinking mass spectrometry data from wild-type OMCCs to evaluate CagX intraprotein crosslinks. We identified 261 total (78 unique) intraprotein crosslinks involving the CagX PI domain in the wild-type OMCCs; 27 unique lysine pairs within 30 Å (71) were mapped into the AlphaFold 3 prediction of the CagX PI (Fig. 8; Table S4). These crosslinking results are consistent with the AlphaFold 3 prediction of the CagX PI structure. The relatively large number of intraprotein crosslinks involving the CagX PI domain is contrasted by the absence of interprotein crosslinks involving the CagX PI domain (Fig. 7).

## DISCUSSION

VirB9 proteins are important components of bacterial T4SSs (Fig. S1) (12, 43, 50, 63). *H. pylori* CagX, a VirB9 homolog (3, 10, 12), contains regions that are structurally similar to other VirB9 proteins, as well as a 130-amino-acid region called the periplasmic insertion (PI) (12) that is specific to *H. pylori*. In this study, we investigated the contributions of conserved VirB9 domains and the *H. pylori*-specific PI domain to the activity and overall organization of the Cag T4SS OMCC. Through construction and analysis of mutant *H. pylori* strains producing variant forms of CagX, we show that each of the three CagX domains (PR, OMC, and PI regions) is required for Cag T4SS activity. We were unable to isolate stable OMCCs from strains engineered to produce only the CagX OMC domain (CagX Δ1–336; CDO1) or only the PR domain (CagX Δ337–522; CDO2), but strains producing a Split CagX (PR and OMC domains produced as two separate proteins; CDO3) or CagX ∆131–260 (deletion of the species-specific PI region; CDO4) were capable of forming partially intact OMCCs.

Analysis of OMCCs from the strain producing Split CagX revealed assembly of the Cag T4SS PR subassembly of the OMCC (consisting of 17 copies of the PR domains of CagX and CagY) (Fig. 3; Table S5; Table 2). Analysis of OMCCs from the strain producing CagX ∆131–260 (deletion of the species-specific PI region) revealed an assortment of particles (Fig. 5; Fig. S8), consisting of CagX and CagY, as well as other proteins known to be OMCC components (CagM, CagT, and Cag3) (Table 2). Notably, the levels of CagT and Cag3 were markedly reduced in OMCC preparations from the CagX ∆131–260 mutant compared to levels of CagT and Cag3 in OMCC preparations from the wild-type strain.

To investigate spatial relationships between CagX and other OMCC components, we performed crosslinking mass spectrometry experiments. In previous studies, crosslinking mass spectrometry has been used to investigate the spatial proximity of components of several types of secretion systems, as well as spatial relationships between secreted proteins and secretion system components (72–76). In the current study, crosslinking mass spectrometry analysis of wild-type OMCCs yielded interprotein crosslinks that were consistent with the known localization of individual proteins in the OMCC structure (determined by single-particle cryo-EM analyses) and known protein-protein interactions. Beyond providing corroborating evidence for the maps generated by previous single-particle cryo-EM analyses, crosslinking mass spectrometry provided insights into protein regions that were not resolved in the previous cryo-EM analyses. For example, about 50% of CagM, 80% of the N-terminus of CagY, a loop region that forms part of the CagY antenna projection (AP) predicted to insert into the outer membrane (62), and the CagX periplasmic insertion (PI) were unresolved in the previous Cag T4SS OMCC cryo-EM structures (12). In the current study, we detected multiple CagY-CagM interprotein crosslinks (Fig. 6), providing evidence for the physical proximity of the crosslinked amino acids in CagY and CagM that were not visible in the EM density maps.

In parallel with crosslinking mass spectrometry analysis of wild-type OMCCs, we conducted the same crosslinking mass spectrometry analysis on OMCCs formed by the CagX ∆131–260 mutant and thereby investigated potential similarities and differences in assembly states of the wild-type and mutant complexes. The number of CagX-CagY and CagX-CagM interprotein crosslinks detected in OMCCs from the CagX ∆131–260 mutant was greater than the number of corresponding crosslinks detected in wild-type OMCCs (Fig. 7). We speculate that the sites of these crosslinks in the mutant OMCCs were more accessible to the crosslinker than corresponding sites in the wild-type OMCCs, due to the loss of Cag3 and CagT from many of the particles isolated from the CagX ∆131–260 mutant and the resulting structural heterogeneity of the OMCCs. In addition, we speculate that the CagX ∆131–260 protein might adopt a broader range of conformational states and may be less rigidly structured than the wild-type protein, which could lead to an increased number of crosslinking events. Most importantly, the crosslinking mass spectrometry analysis revealed that the CagX ∆131–260 mutant protein (lacking the PI domain) retains the capacity to interact with both CagY and CagM, which suggests that the CagX ∆131–260 protein (or at least a portion of this protein) retains structural integrity (Fig. 7).

The CagX PI is an *H. pylori* species-specific domain, consisting of 130 amino acids, which has not been resolved by cryo-EM (12). AlphaFold 3 (68) predicts that the CagX PI domain consists of two α-helices (Fig. 4C). This model of the CagX PI structure is supported by the analysis of CagX intraprotein crosslinks in the current study (Fig. 8A; Table S4). One of the α-helices within a single asymmetric unit of the predicted CagX PI structure is hydrophilic, while the other is hydrophobic (Fig. 8B). Further studies will be needed to determine the precise localization of the CagX PI domain within the OMCC.

While the PI domain is not found in VirB9 proteins of minimized bacterial T4SSs, analogous structures might exist in other expanded T4SSs. For example, the N-terminal periplasmic region of *L. pneumophila* DotH/IcmK (residues 104–143) folds into α-helices and also includes a region of about 100 residues that has not been resolved by cryo-EM analysis (Fig. S1B). We speculate that the modified VirB9 proteins in these expanded T4SSs may have direct or indirect roles in facilitating the assembly of OMCCs containing species-specific components not found in minimized T4SSs. Consistent with this hypothesis, we show that the CagX PI domain is required for stable association of Cag3, CagT, CagW, CagH, CagI, and CagL with the Cag T4SS OMCC. The CagX PI domain might stabilize the Cag T4SS OMCC by forming a connection between the PR region of the OMCC and the stalk. Alternatively, the modified VirB9 proteins in *H. pylori* and *L. pneumophila* may have specialized roles in substrate recruitment.

CagW, CagH, CagI, and CagL have been reported to associate with the Cag T4SS OMCC (51), and all four of these proteins are required for Cag T4SS activity (51, 52). These four proteins do not exhibit any unambiguous relatedness to components in T4SSs from other bacterial species and are present in relatively low abundance in preparations of isolated OMCCs. The precise localization of these four proteins relative to the rest of the OMCC remains unknown. In the current study, we found that an intact CagX protein, including the PR, OMC, and PI domains, is required for stable association of CagW, CagH, CagI, and CagL with the Cag T4SS OMCC. In our current experiments using the crosslinker BS3, we did not detect interprotein crosslinks involving the low abundance proteins CagW, CagH, CagI, and CagL, limiting insight into the localization of these proteins relative to other OMCC components. The absence of detectable crosslinks involving CagW, CagH, CagI, and CagL is probably attributable to the relatively low abundance of these proteins in the OMCC preparations (51, 52). In addition, since BS3 crosslinks primary amine residues, protein regions with a limited number and accessibility of lysine residues may not be efficiently crosslinked with the current approach.

A model for Cag T4SS assembly proposes that the OMCC assembles prior to assembly of the inner membrane complex (11). Previous analysis of OMCCs from a mutant lacking the CagY AP, a region proposed to anchor Cag T4SS OMCCs into the outer membrane, found that the CagY AP is not required for OMCC assembly (62). We have also previously reported that *H. pylori* Δ*cagT* and Δ*cagM* mutants can assemble OMCCs in which the Cag T4SS PR is structured and the OMC is unstable (61), similar to OMCCs purified from the Split CagX mutant strain in this study. Collectively, these findings suggest that the PR is more stable than the OMC under the tested conditions and suggest that the oligomerization of CagX and CagY to form the PR might be a key step in OMCC assembly.

A recent expansion of the model for Cag T4SS OMCC assembly proposes that CagX, CagY, and CagM first assemble into a central cylinder, followed by the interaction between CagX and the C-terminus of a copy of CagT, prior to subsequent recruitment of CagT-Cag3 dimers and Cag3 dimers or trimers to the OMCC (77). Previous cryo-EM analyses of a ∆*cag3* mutant reported the assembly of stable Cag T4SS OMCCs (lacking five copies of Cag3 and a copy of CagT) (12), which supports the proposed model. In the current study, we show OMCCs from a strain lacking the CagX periplasmic insertion have heterogeneous morphologies. These could represent varying stages of OMCC assembly or might represent OMCC structures at various stages of disassembly. Crosslinking mass spectrometry analysis showed numerous crosslinks between CagX ∆131–260 and CagM, which suggests that CagX-CagM interactions are maintained despite relative instability of OMCCs formed by the CagX ∆131–260 mutant. Collectively, these results suggest that the CagX periplasmic insertion domain is required for the stability of protein-protein interactions of the central cylinder (CagX-CagY-CagM) with the peripheral OMC components CagT and Cag3 (Fig. 6 and 7).

In summary, at the outset of this study, it was known that CagX was a key component of the Cag T4SS OMCC, but no structure-function analyses had been attempted for this protein. In this study, we show that two conserved regions of CagX (OMC and PR domains) are each required for Cag T4SS activity and assembly of an intact OMCC, consistent with expectations. We show that a species-specific domain of CagX (the PI domain) is also required for Cag T4SS activity, but the CagX ∆131–260 protein (lacking the PI domain) retains the ability to form partially intact OMCCs. We show that the CagX PI domain is required for stable association of CagT and Cag3 with the OMC subassembly, as well as the association of CagW, CagH, CagI, and CagL with the OMCC. In addition, we show that crosslinking mass spectrometry can be used as a powerful method to define spatial relationships among components of the T4SS for which the structures have not yet been successfully resolved by cryo-EM, and we use this method to demonstrate spatial proximity of both the wild-type CagX and CagX ∆131–260 proteins to CagY and CagM.

In future studies, it will be important to further elucidate mechanisms by which the incorporation of the species-specific periplasmic insertion domain into CagX confers specialized structural and functional properties to the *H. pylori* Cag T4SS. The Cag T4SS is required for the delivery of both CagA and non-protein substrates (LPS metabolites, peptidoglycan, and DNA) into host cells; hence, it will be important to investigate how specialized properties of CagX might contribute to substrate selection and translocation of each of these substrates. For example, future studies could define the contributions of the lengths and amino acid sequences of the CagX PI and flexible linker regions to Cag T4SS function in secreting different types of substrates. In addition, it will be important to investigate how species-specific features of additional components of the Cag T4SS contribute to T4SS assembly and activity.

## MATERIALS AND METHODS

### Bacterial strains

*H. pylori* strains generated and used in this study are listed in Table 1. *H. pylori* strains producing several modified forms of conserved CagX domains were generated by mutating the chromosomal *cagX* locus. To do this, plasmids containing modified *cagX* sequences, encoding CagX ∆1–336 (CDO1), CagX ∆337–522 (CDO2), and Split CagX (CDO3), along with flanking sequences and a chloramphenicol resistance gene, were designed and synthesized (see details in Fig. S2). DNA sequences were cloned into the pUC57 vector (GenScript). *H. pylori* strain SCT72 (modified to express a hemagglutinin [HA] epitope-tagged CagF; Table 1) was transformed with these plasmids, and chloramphenicol-resistant colonies were selected. The CagX numbering system is based on designating methionine (the first CagX amino acid) as amino acid number 1. Sequences encoding the signal peptide of the vacuolating cytotoxin A gene (*vacA*) were fused to CagX ∆1–336 and the C-terminal portion of the Split CagX construct (Fig. S2) to facilitate the secretion of the proteins across the inner membrane.

To generate a strain producing CagX ∆131–260 (CDO4), a plasmid was designed and synthesized to replace the *rdxA* locus (*hp0954* in *H. pylori* strain 26695) with a sequence encoding CagX ∆131–260, conferring metronidazole resistance to the bacterial transformants. The plasmid also contained a copy of the endogenous *cagV-cagY* operon promoter and a modified *cagX* ribosomal binding site, inserted upstream of the modified *cagX* gene (Fig. S2). The DNA sequence was cloned into the pUC57 vector (GenScript) and introduced into the HA-CagF Δ*cagX* strain (that lacks the endogenous *cagX* gene and produces HA-tagged CagF; Table 1).

All plasmids were transformed into *E. coli* strain DH5α and grown on either Luria-Bertani (LB) agar plates supplemented with ampicillin (100 µg/mL) or in Terrific Broth supplemented with ampicillin (100 µg/mL). Plasmid preparations were generated using a commercially available kit (Macherey-Nagel) and then introduced into *H. pylori* by natural transformation. The engineered *H. pylori* mutant strains described did not exhibit any detectable growth defects, based on the analysis of optical density.

### *H. pylori* culture conditions

*H. pylori* strains used in this study were grown on trypticase soy agar plates containing 5% sheep blood. *H. pylori* strains were inoculated into bisulfite-free *Brucella* broth liquid culture (containing tryptone, peptone, yeast extract, dextrose, and sodium chloride) with 10% heat-inactivated fetal bovine serum (FBS), shaking at 220 rpm in a 37°C incubator supplied with 5% CO_2_. *H. pylori* mutant strains were selected in the presence of chloramphenicol (2.5 µg/mL), gentamicin (5 µg/mL), or metronidazole (7.5 µg/mL).

### CagA translocation assays

CagA translocation assays were performed as described previously (66). AGS cells were cultured in RPMI media supplemented with 10% heat-inactivated FBS at 37°C with supplemental 5% CO_2_. AGS cells were seeded at 2.5 × 10^5^ cells/mL in six-well plates and cultured for 24 h. AGS cells were infected with the indicated *H. pylori* strain at a multiplicity of infection (MOI) of 100:1 for 4 h. AGS and *H. pylori* cells were lysed with buffer containing 50 mM Tris-HCl (pH 7.4), 150 mM NaCl, 1% NP-40, 2 mM sodium orthovanadate, protease inhibitor cocktail (cOmplete Tablets, Mini EDTA-free, Roche), and a phosphatase inhibitor cocktail tablet (PhosSTOP, Roche). Protein concentrations were measured by Pierce BCA assay (Thermo Scientific) and standardized prior to resolving by SDS-PAGE on a 7.5% acrylamide gel (Bio-Rad) and transferring to 0.22 μm nitrocellulose membrane (Li-Cor). Phosphorylated CagA was detected by immunoblotting with a phosphotyrosine mouse monoclonal antibody (anti-PY99; Santa Cruz Biotechnology). Membranes were stripped and re-probed for CagA using an anti-CagA rabbit polyclonal antiserum (67).

### IL-8 ELISA

AGS cells were seeded in 96-well plates at 2.5 × 10^5^ cells per well in RPMI media supplemented with 10% heat-inactivated FBS and incubated at 37°C with supplemental 5% CO_2_. After 24 h, AGS cells were infected with the indicated *H. pylori* strains at an MOI of 100:1 for 4 h. Supernatants from co-cultures were collected and used for an enzyme-linked immunosorbent assay (ELISA) to quantify levels of human IL-8/CXCL8 (Biotechne R&D Systems) following the manufacturer’s protocol.

### Immunoprecipitation

*H. pylori* strains were cultured in 125 mL *Brucella* broth media supplemented with 10% FBS for about 24 h to a final optical density at 600 nm (OD_600_) of approximately 0.6–1.0. Cells were harvested by centrifugation and lysed in a radioimmunoprecipitation assay (RIPA) buffer (200 mM HEPES, 300 mM NaCl, 1% NP-40, 0.25% sodium deoxycholate, at pH 7) with 1 mM phenylmethylsulfonyl fluoride (PMSF) and protease inhibitor cocktail (cOmplete Tablets, Mini EDTA-free, Roche). Cells were lysed by sonication on ice at 25% amplitude, with 4 rounds of 10 s pulses on/off, then rocked for 1 h at 4°C. Soluble protein fraction was obtained by centrifugation at 12,000 × *g* for 15 min. Protein G dynabeads (Invitrogen) were washed and non-covalently bound with mouse monoclonal anti-HA antibodies for 15 min prior to incubation with clarified bacterial lysates for 30 min at room temperature. Flowthrough was discarded, and beads were washed thrice in RIPA buffer with a reduced concentration of sodium deoxycholate (0.025%). Protein complexes were eluted from anti-HA conjugated beads by addition of 200 µg/mL HA peptide (GenScript) in RIPA wash buffer.

### Coomassie stain analysis

Purified proteins were resolved by SDS-PAGE using 4%–20% gradient gels (Bio-Rad). Resolved protein gels were stained with Simply Blue Safe Stain (Invitrogen) for 1 h and destained with water.

### Immunoblot analysis

Protein bands were resolved by SDS-PAGE using 4%–20% gradient gels (Bio-Rad) and transferred to 0.22 μm nitrocellulose membrane (Li-Cor). Membranes were immunoblotted using rabbit polyclonal antiserum (anti-CagX, anti-CagY, anti-Cag3 (67), or anti-CagT (78)) or mouse monoclonal antibody (anti-HA [CagF] 12CA5) (51, 62), followed by peroxidase-conjugated secondary antibodies.

### Negative stain electron microscopy

Purified protein complexes were adsorbed onto glow-discharged, 400-mesh copper grids covered with formvar carbon film (Electron Microscopy Sciences). Grids were washed in water and stained with 0.75% uranyl formate. Negative stain images were taken using a Tecnai Spirit T12 transmission electron microscope (Thermo Fisher Scientific) operated at 120 kV and at a nominal magnification of 26,000x (2.09 Å/pixel). Images were acquired with SerialEM on a 4K × 4K Rio complementary metal-oxide semiconductor camera (Gatan) at −1.5-μm defocus value.

### Negative stain EM image processing

For Split CagX, 750 micrographs were collected and imported into cryoSPARC (v. 4.7.1) for 2D class averaging (79). 156 reference particles were manually picked from a subset of 29 micrographs and used to train a Topaz model (80). Topaz particle picking resulted in a total of 12,742 particles. After extracting particles with a 300 pixel box size (2.09 Å/pixel), 2D classification was performed using cryoSPARC.

For CagX ∆131–260, 2,112 micrographs were collected and imported into cryoSPARC (v. 4.7.1) for 2D class averaging; 882 reference particles were manually picked from a subset of 66 micrographs and used as references for template picker. Template particle picking in cryoSPARC resulted in a total of 68,809 particles. After extracting particles with a 400 pixel box size (2.09 Å/pixel), 2D classification was performed to remove junk particles, resulting in a final particle count of 35,008 particles. The final stack of particles was used for 2D classification using cryoSPARC.

### Tandem mass spectrometry analysis

To analyze the protein content of the immunopurified samples, tryptic peptides were prepared using S-Trap (Protifi), according to the manufacturer’s recommended protocol. Peptides were resuspended in 40 µL 0.2% formic acid with 0.015% n-Dodecyl-β-D-maltoside (DDM). Data-dependent acquisition with parallel accumulation-serial fragmentation (DDA-PASEF) data were acquired using a 30-min aqueous to organic gradient method delivered via a nanoELUTE2 on a PepSep microbore column (75 µm internal diameter, 25 cm length, and 1.5 µm particle size) coupled to a timsTOF HT (Bruker) using a 10 µm Captive Spray emitter. DDA-PASEF data were collected in 5 PASEF ramps from 0.7 to 1.3 1/k0 covering m/z windows from 300 to 1,700 with 100 accumulation times in the TIMS cell. Peptide MS/MS spectra were queried against an *H. pylori* protein database (strain 26695) (81) using FragPipe 19 with default parameters (82), filtered using the included implementation of Percolator to 0.1% false discovery rate (FDR), and then visualized using Scaffold 5 (Proteome Software).

### Crosslinking mass spectrometry analysis

OMCCs were immunoprecipitated from the indicated *H. pylori* strains using HA-CagF as a bait, as previously described, with minor modifications: *H. pylori* strains were cultured in 500 mL *Brucella* broth media supplemented with 10% FBS to a final OD_600_ of about 0.8–1.0, and harvested cells were lysed with five rounds of sonication. Purified protein complexes were ultrafiltered to a final volume of about 300 µL by centrifugation at 5,000 × *g* for 25 min into a buffer without detergent (200 mM HEPES, 300 mM NaCl, 1 mM PMSF, pH 7) using Amicon Ultra-15, 10-kDa cutoff filter tubes (Sigma-Aldrich) to remove excess HA peptide used in the elution step; 80 μL of ultrafiltered protein complexes were aliquoted and incubated with varying concentrations of BS3 crosslinker (Thermo Scientific) (0 mM [control], 0.1 mM, or 0.25 mM) in a volumetric ratio of 5:1 (purified protein:crosslinker) for 30 min at room temperature with end-on-end rotation. The crosslinking reaction was quenched by the addition of 50 mM Tris-HCl (pH 8) for 15 min at room temperature with end-on-end rotation. BS3-treated samples and controls were analyzed by SDS-PAGE and Coomassie staining as previously described.

Samples were trypsin-digested via S-trap and analyzed by data-dependent (DDA) LC tandem mass spectrometry using an Ultimate 3000, an 18 cm by 100 µm lab-packed reversed phase column (Phenomonex Jupter, 3-µm particle, 300 Å pore size) resolved over a 90-min gradient with data collected on a ThermoFisher Exploris 240. Data were acquired for peptides over a 350–1,500 m/z range, with the top 20 peaks triggering MS/MS, filtering for charge states 1–6, MIPS filtering on, and dynamic exclusion set to 20 s.

Raw mass spectrometry files were further analyzed using the pLink 3 software (69) to identify the sites of BS3 interprotein crosslinks, between peptides from two different proteins, and intraprotein crosslinks, within peptides from the same protein. Network maps of compiled crosslinking data were depicted using the xiNET cross-link viewer (70).

## SUPPLEMENTAL MATERIAL

Supplemental figures iai.00699-25-s0001.pdf Fig. S1 to S9.

Supplemental tables iai.00699-25-s0002.pdf Tables S1 to S4.

ASM does not own the copyrights to Supplemental Material that may be linked to, or accessed through, an article. The authors have granted ASM a non-exclusive, world-wide license to publish the Supplemental Material files. Please contact the corresponding author directly for reuse.
