# Supplementary material for: Contributions of conserved and species-specific CagX (VirB9) domains to the assembly and function of the Helicobacter pylori Cag type IV secretion system
Source: Infect Immun. 2026 Jun 10;94(7):e00699-25. doi: 10.1128/iai.00699-25 (PMC13417560; doi:10.1128/iai.00699-25)
Supplement: Supplemental figures — Fig. S1 to S9. [file iai.00699-25-s0001.pdf]

SUPPLEMENTAL FIGURES

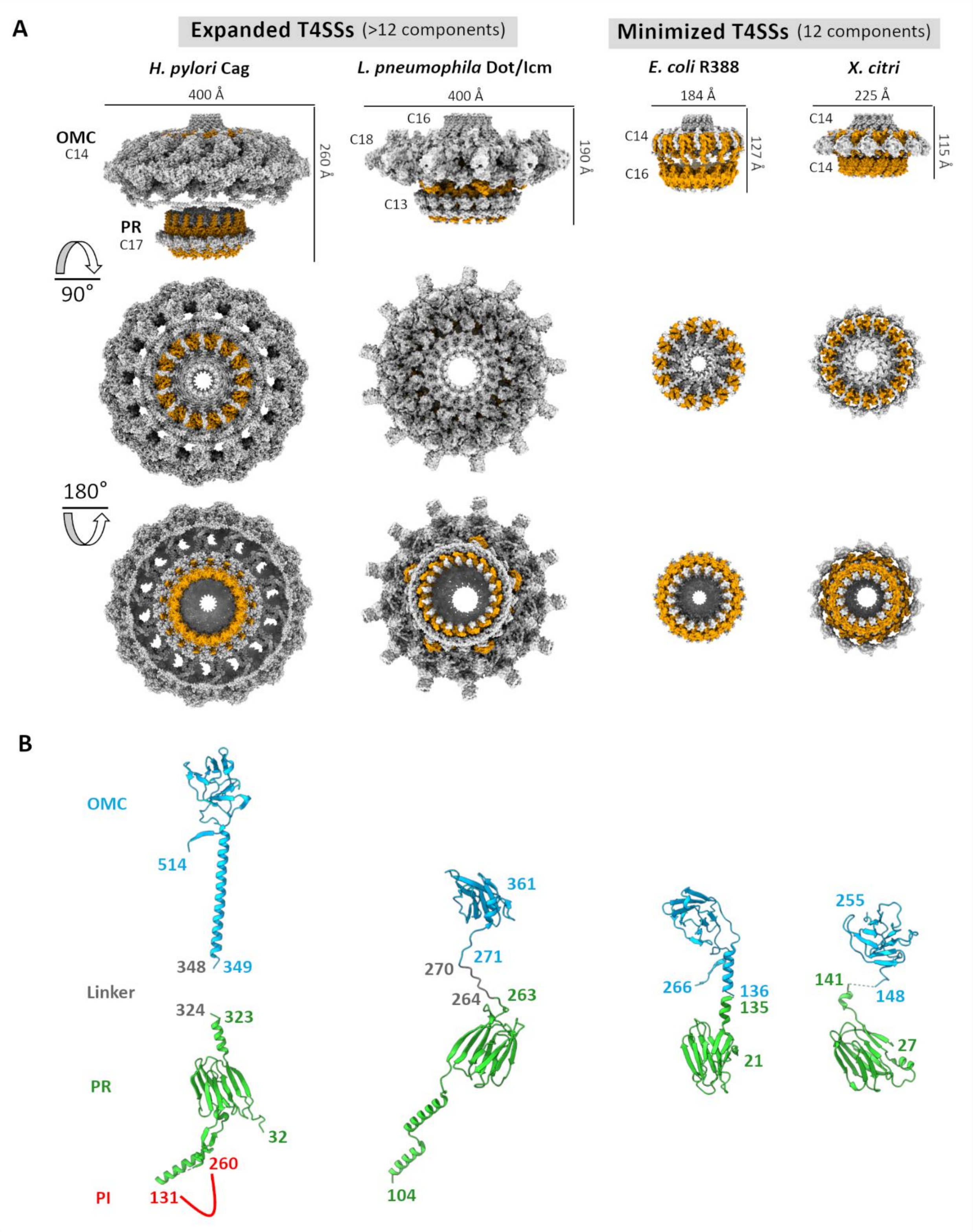

Figure S1. Structural comparisons of *H. pylori* CagX to VirB9 proteins in T4SSs from other bacterial species. (**A**) VirB9 homologs (orange) are a major structural component of bacterial T4SSs. The top panel shows side views of expanded and minimized T4SS OMCCs. Expanded T4SS OMCCs: *H. pylori* (PDB: 6X6S and 6X6J) and *Legionella pneumophila* (PDB: 7MUY). Minimized T4SSs OMCCs: *Escherichia coli* R388 plasmid (PDB: 8RT4 and 8RT5) and *Xanthomonas citri* (PDB: 6GYB). Bacterial T4SSs OMCCs are rotated 90°and 180°, showing views looking from the outer- to inner- membranes (middle panel) or from inner- to outer-membranes (bottom panel). Symmetries of various regions of the OMCCs are labeled. (**B**) Structural comparison of one asymmetric unit of CagX (left) with other VirB9 homologs reveals two conserved domains - the outer membrane cap (OMC; blue) and periplasmic ring (PR; green) – and a linker region (grey). The site of an *H. pylori*-specific periplasmic insertion (PI; red) domain is shown as a red loop. Cryo-EM densities for the CagX linker region and PI domain were not visible in the density maps. CagX is larger than VirB9 proteins from other bacterial species (*H. pylori* CagX: 522 residues; *L. pneumophila* DotH/IcmK: 361 residues; *E. coli* TwrF: 266 residues; *X. citri* VirB9: 255 residues).

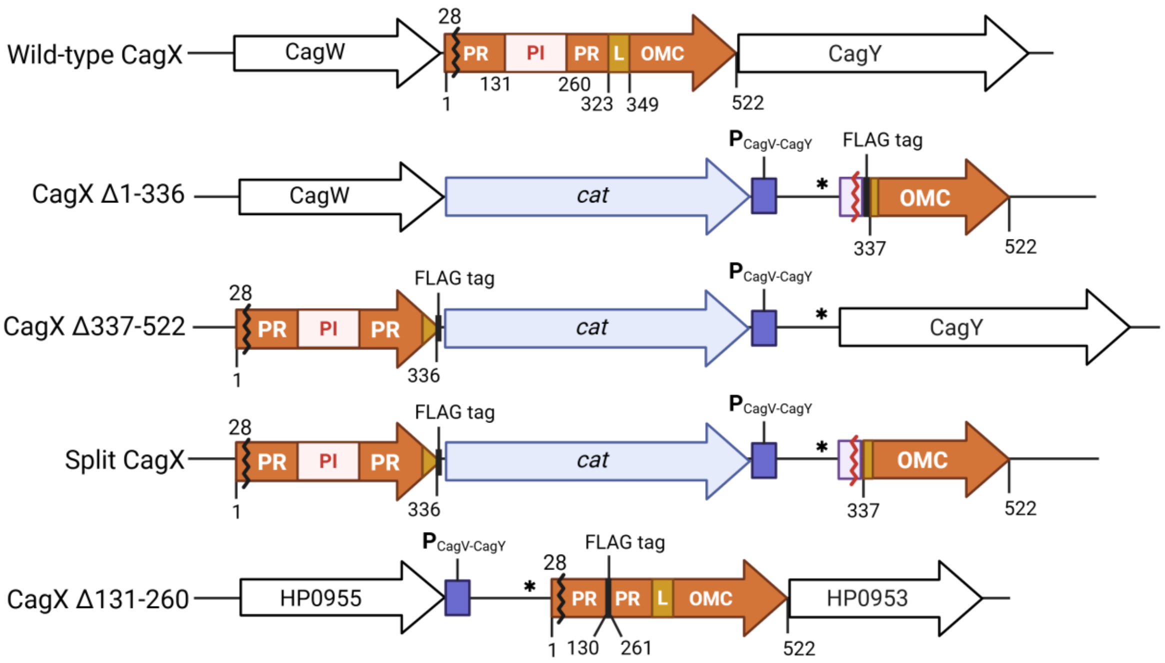

Figure S2. Schematics of DNA constructs used to generate the indicated CagX mutant strains - CagX Δ1-336 (CDO1), CagX Δ337-522 (CDO2), Split CagX (CDO3), and CagX Δ131-260 (CDO4). *cat*, chloramphenicol acetyltransferase gene of *Campylobacter coli*; $P_{CagV\text{-}CagY}$, promoter for the CagV – CagY operon in *H. pylori* strain 26695; *, ggagga - modified Shine-Dalgarno sequence of the

CagV-CagY operon. Sequences encoding FLAG tags were also incorporated into the constructs, as indicated. This numbering system includes the CagX signal sequence (residues 1 – 28, black zig-zag line; based on SignalP 6.0 prediction). Signal sequence for the VacA toxin (residues 1-33, red zig-zag line) was inserted upstream of the sequences encoding the CagX OMC domain to ensure protein secretion across the inner membrane. PR, periplasmic ring. PI periplasmic insertion domain. L, linker. OMC, outer membrane cap.

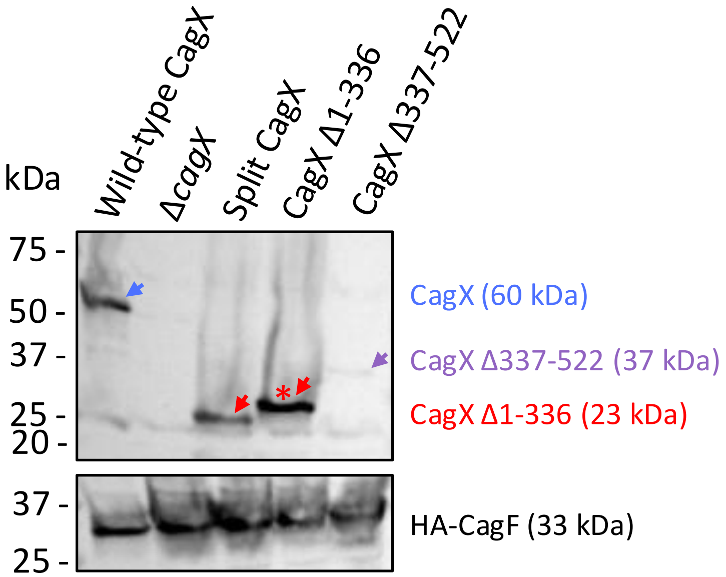

Figure S3. Top panel: *H. pylori* lysates from CagX Δ1-336 (CDO1), CagX Δ337-522 (CDO2), and Split CagX (CDO3) were probed with CagX antiserum and compared with the wild-type CagX strain (HA-CagF 26695; positive control) and a *ΔcagX* mutant (HA-CagF *ΔcagX*; negative control), each modified to produce an HA-CagF protein. Bottom panel: Immunoblot for HA-CagF was used as a loading control. Immunoreactive bands corresponding to the CagX OMC (CagX Δ1-336 domain; red arrowheads) are visible in lysates of the Split CagX mutant and the CagX Δ1-336 mutant, but not the CagX Δ337-522 mutant lacking the OMC domain, as expected. Red asterisk (*) – The higher molecular mass for the band corresponding to the CagX OMC domain in the CagX Δ1-336 mutant is accounted for by the presence of a FLAG tag (Fig. S2). An immunoreactive band corresponding to the CagX PR (CagX Δ337-522; purple arrowhead) is weakly detected in lysate from the CagX Δ337-522 mutant and not visible in lysate from the Split CagX mutant. See corresponding mass spectrometry analyses of immunopurified samples shown in Fig. S4.

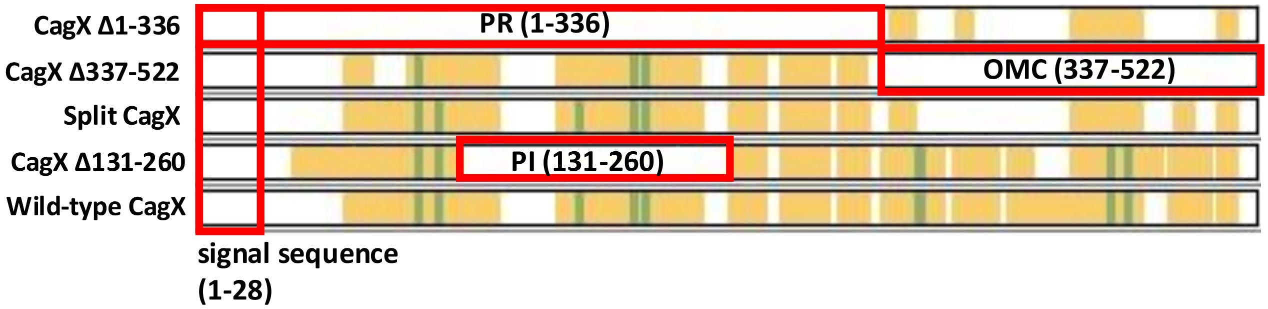

Figure S4. Tandem mass spectrometry peptide sequence coverage for CagX immunoprecipitated from the indicated strains (left) are consistent with the expected results, based on experimental modifications to CagX. Total number of spectral counts for CagX from each strain is listed in Table 2.

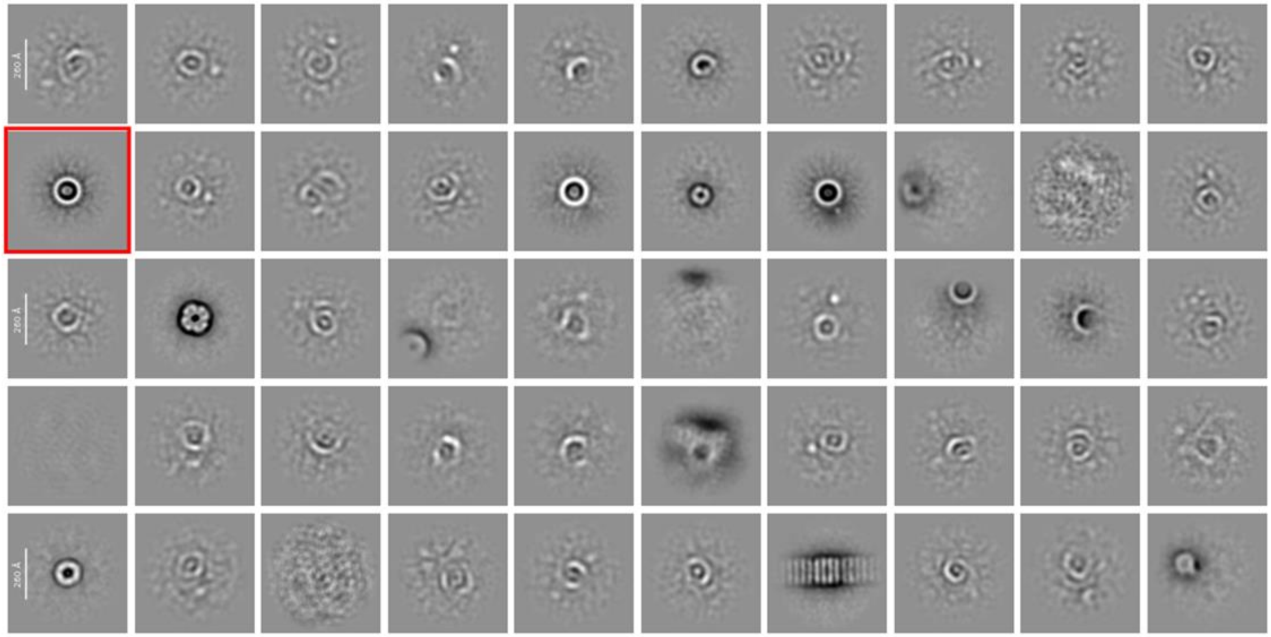

Figure S5. Negative stain EM 2D class averages of OMCCs immunoprecipitated from the Split CagX mutant (CDO3) reveals assembly of the Cag T4SS PR (17 copies of CagX and CagY). Red rectangle indicates 2D average shown in Fig. 3D.

A

```
VirB9_Agrobacterium     GNYLFFKASQVL------------------------------------------------    93
CagX                    SNHIFIQPKSVKSNL--MFEKEAVNFA----LMTRDYQEFLKTKKLIVDAPDPKELEEQK   145
VirB9_Xanthomonas       ENVFYLKPKNVDVDTNMMIRTATHSYILELKVVATDWQRLEQAKQAGVQ-----------   125
                         * :::: ..*

VirB9_Agrobacterium     ------------------------------------------------------------    93
CagX                    KALEKEKEAKEQAQKAQKDKREKRKEERAKNRANLENLTNAMSNPQNLSNNKNLSEFIKQ   205
VirB9_Xanthomonas       ------------Y-----------------------------------------------   126

VirB9_Agrobacterium     ---------------------------------------------------------TPQ    96
CagX                    QRENELDQMERLEDMQEQAQANALKQIEELNKKQAEETIKQRAKDKINIKTDKPQKSPED   265
VirB9_Xanthomonas       ------------------------------------------------------------   126
```

B

```
J166           DYQEFLKTKKLIVDAPDPKELEEQKKALEKEKEAKEQAQKAQKDKREKRKEERAKNRANL
B8             DYQEFLKTKKLIVDAPDPKELEEQKKALEKEKEAKEQAQKAQKDKREKRKEERAKNRANL
J99            DYQEFLKTKKLIVDAPDPKELEEQKKALEKEKEAKEQAQKAQKDKREKRKEERAKNRANL
PMSS1          DYQEFLKTKKLIVDAPDPKELEEQKKALEKEKEAKEQAQKAQKDKREKRKEERAKNRANL
SS1            DYQEFLKTKKLIVDAPDPKELEEQKKALEKEKEAKEQAQKAQKDKREKRKEERAKNRANL
26695          DYQEFLKTKKLIVDAPDPKELEEQKKALEKEKEAKEQAQKAQKDKREKRKEERAKNRANL
G27            DYQEFLKTKKLIVDAPDPKELEEQKKALEKEKEAKEQAQKVQKDKREKRKEERAKNRANL
           121 ****************************************.******************* 180

J166           ENLTNAMSNPQNLSNNKNLSEFIKQQRENELDQMERLEDMQEQAQANALKQIEELNKKQA
B8             ENLTNAMSNPQNLSNNKNLSEFIKQQRENELDQMERLEDMQEQAQANALKQIEELNKKQA
J99            ENLTNAMSNPQNLSNNKNLSELIKQQRENELDQMERLEDMQEQAQANALKQIEELNKKQA
PMSS1          ENLTNAMSNPQNLSNNKNISEFIKQQRENELDQIERLEDMQEQAQANALKQIEELNKKQA
SS1            ENLTNAMSNPQNLSNNKNISEFIKQQRENELDQIERLEDMQEQAQANALKQIEELNKKQA
26695          ENLTNAMSNPQNLSNNKNLSEFIKQQRENELDQMERLEDMQEQAQANALKQIEELNKKQA
G27            ENLTNAMSNPQNLSNNKNLSELIKQQRENELDQMERLEDMQEQAQANALKQIEELNKKQA
           181 *****************:**:***********:************************** 240

J166           EEAVRQRAKDKINIKTDKSQKSPEDNSIELSPSDSAWRTNLVVRTNKALYQFILRIAQKD
B8             EEAVRQRAKDKINIKTDKSQKSPEDNSIELSPSDSAWRTNLVVRTNKALYQFILRIAQKD
J99            EEAVRQRAKDKISIKTDKSQKSPEDNSIELSPSDSAWRTNLVVRTNKALYQFILRIAQKD
PMSS1          EETIKQRAKDKISIKTDKPQKSPEDNSIELSPSDSAWRTNLVVRTNKALYQFILRIAQKD
SS1            EETIKQRAKDKISIKTDKPQKSPEDNSIELSPSDSAWRTNLVVRTNKALYQFILRIAQKD
26695          EETIKQRAKDKINIKTDKPQKSPEDNSIELSPSDSAWRTNLVVRTNKALYQFILRIAQKD
G27            EETIKQRAKDKISIKTDKSQKSPEDNSIELSPSDSAWRTNLVVRTNKALYQFILRIAQKD
           241 **:::*******.*****.***************************************** 300
```

Figure S6. (**A**) Amino acid sequence alignment of *H. pylori* CagX with VirB9 from the minimized T4SSs of *Agrobacterium tumefaciens* and *Xanthomonas citri*, showing the *H. pylori*-specific PI domain (highlighted in yellow). (**B**) The CagX PI (highlighted in yellow) is conserved across *H. pylori* strains. Amino acid numbering system is based on *H. pylori* strain 26695.

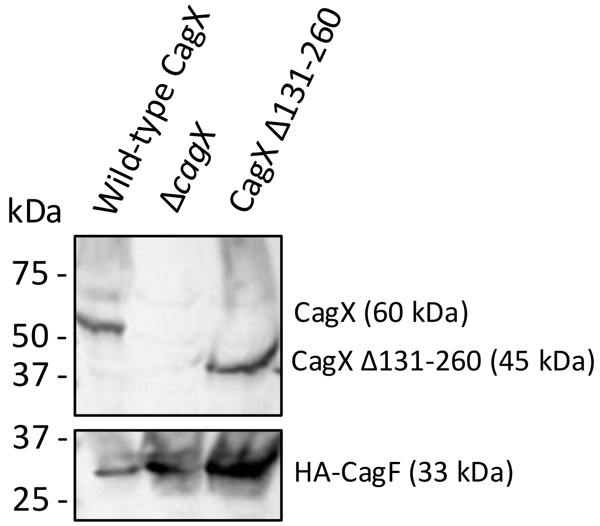

Figure S7. Top panel: Immunoblot of *H. pylori* lysates using CagX antibodies show that a CagX protein of the expected mass is produced by the CagX Δ131-260 mutant strain (CDO4). Bottom panel: Immunoblot for HA-CagF was used as a control.

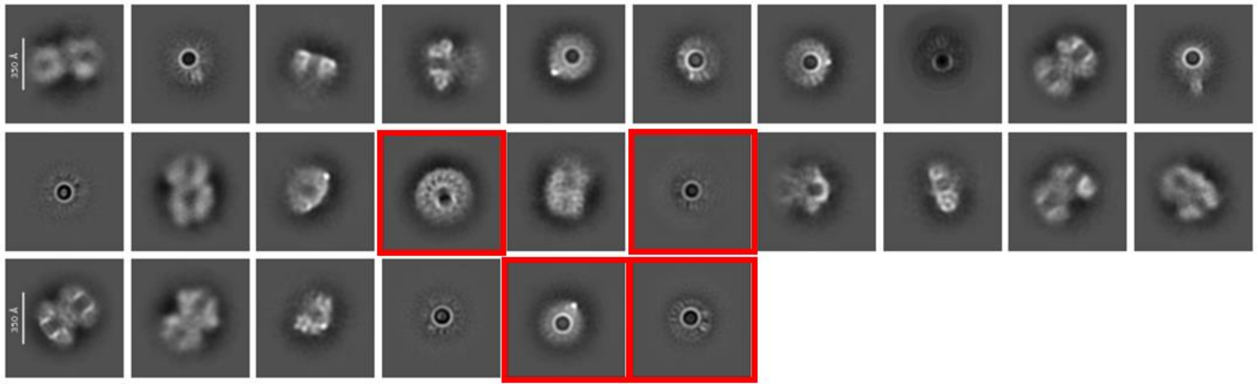

Figure S8. Negative stain EM 2D class averages of partially assembled Cag T4SS OMCCs immunoprecipitated from the CagX Δ131-260 mutant (CDO4), consisting of the 17-fold symmetric PR and various assemblies of the OMC. Red rectangles indicate 2D averages shown in Fig. 5D.

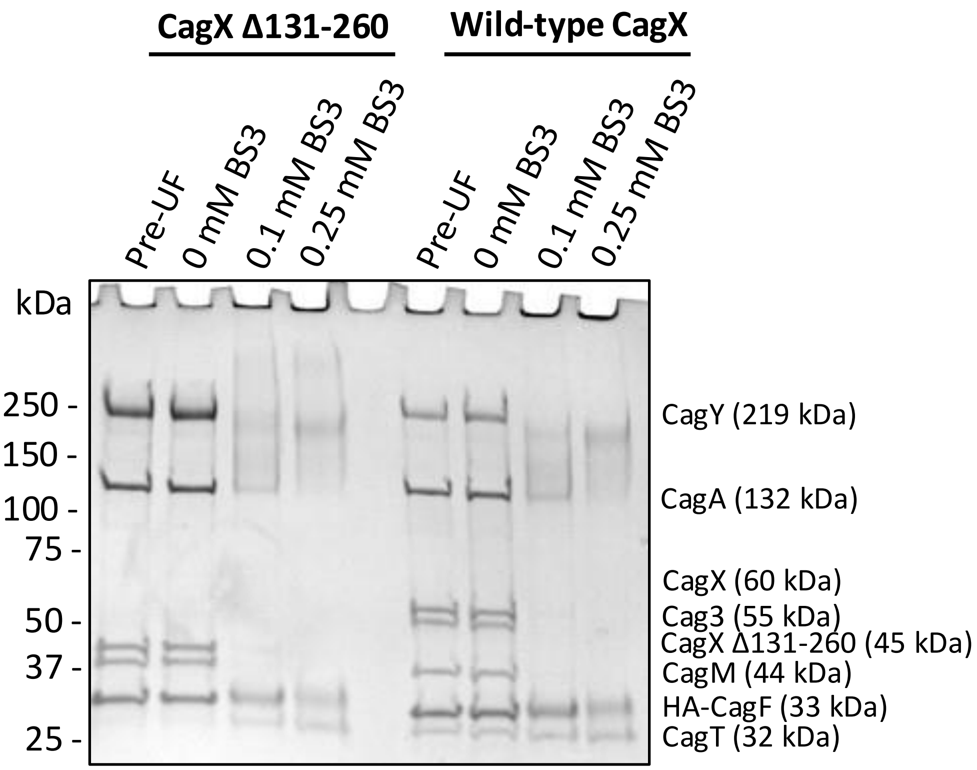

Figure S9. Coomassie-stained gel of OMCCs first purified from CagX Δ131-260 (CDO4) and wild-type (HA-CagF 26695) strains then treated with BS3 crosslinker at 0 mM (negative control), 0.1 mM or 0.25 mM. Pre-UF (pre-ultrafiltration): OMCCs eluted with HA peptide prior to ultrafiltration and BS3 crosslinking reaction, as described in Methods.
