# Supplementary material for: Contributions of conserved and species-specific CagX (VirB9) domains to the assembly and function of the Helicobacter pylori Cag type IV secretion system
Source: Infect Immun. 2026 Jun 10;94(7):e00699-25. doi: 10.1128/iai.00699-25 (PMC13417560; doi:10.1128/iai.00699-25)
Supplement: Supplemental tables — Tables S1 to S4. [file iai.00699-25-s0002.pdf]

SUPPLEMENTAL TABLES

Table S1. LC-MS/MS analysis of BS3-treated complexes from CagX Δ131-260 and wild-type strains.

Identified proteins[a]	Wild-type CagX[b]			CagX Δ131-260[b]		
	**0 mM**	**0.1 mM**	**0.25 mM**	**0 mM**	**0.1 mM**	**0.25 mM**
CagF	208	115	70	131	106	70
CagA	1227	923	809	1152	946	764
CagX	473	400	363	398	373	326
CagY	853	633	496	1085	783	565
CagM	280	170	158	286	190	126
CagT	156	125	104	41	32	30
Cag3	583	438	353	114	76	53
CagW	30	25	19	0	0	0
CagH	23	20	18	0	0	0
CagI	37	35	34	1	0	0
CagL	16	14	14	0	0	0
CagE	22	13	8	14	9	4
CagV	12	11	8	10	10	7
CagN	14	14	14	7	10	10
CagG	5	5	6	2	3	4
CagD	1	1	0	2	2	0
No. of Cag Spectra	3732	2827	2404	3112	2434	1889
% Cag Spectra	56%	59%	63%	57%	62%	66%
Total Spectral Counts	6666	4772	3822	5500	3924	2870

[a]Cag proteins were detected by tandem mass spectrometry analysis. Numbers represent spectral counts.

[b]Proteins were isolated by immunoprecipitation of HA-CagF from strains producing Wild-type CagX (HA-CagF 26695) or CagX Δ131-260 (strain CDO4) prior to treatment with the indicated concentration of BS3 crosslinker, as described in the Methods.

Table S2. Interprotein crosslinks detected in BS3-treated wild-type OMCCs[a].

Protein 1	Protein 1 lysine position	Protein 2	Protein 2 lysine position	Total BS3 interprotein crosslinks
CagY	1671	CagX	515	2
CagY	1924	CagX	393	4
CagY	1910	CagX	393	1
CagY	1827	CagX	490	1
CagY	1734	CagX	499	2
CagY	1924	CagX	515	1
CagY	1924	CagX	499	1
CagY	1617	CagX	341	2
CagY	1668	CagX	515	1
CagY	1671	CagX	515	2
CagY	1608	CagX	337	2
CagY	1612	CagX	337	2
CagY	764/1347 [b]	CagX	150	1
CagY	1471	CagX	299	2
CagY	1582	CagX	337	2
CagY	1465	CagX	53	4
CagY	1894	CagX	515	2
CagY	1898	CagX	515	2
CagY	1910	CagX	372	2
CagY	1910	CagX	499	2
CagY	1685	CagX	393	4
CagY	1617	CagX	337	2
CagY	1910	CagX	374	2
CagY	1746	CagX	412	2
CagY	1465	CagX	40	3
CagY	1924	CagX	365	2
CagY	1910	CagX	365	1
CagY	1746	CagX	490	2
CagM	77	CagT	259	1
CagM	170	CagT	179	2
CagM	215	CagT	194	2
CagM	215	CagT	189	1
CagM	215	CagT	259	2
CagM	215	CagT	206	3
CagM	49	CagT	259	3
CagM	215	CagT	200	2
CagM	281	CagT	71	3

CagM	155	CagT	252	2
CagM	236	CagT	252	1
CagM	281	CagT	194	3
CagM	291	CagT	252	2
CagM	100	CagT	243	2
CagM	265	CagT	243	1
CagM	168	CagT	243	2
CagM	236	CagT	129	1
CagM	230	CagT	206	4
CagT	194	CagX	393	3
CagT	194	CagX	515	4
CagT	78	CagX	393	2
CagT	78	CagX	515	2
CagT	78	CagX	499	2
CagT	194	CagX	515	2
CagT	166	CagX	499	1
CagT	145	CagX	490	2
CagT	92	CagX	494	1
CagT	57	CagX	490	2
CagT	57	CagX	494	2
CagT	46	CagX	494	3
CagT	46	CagX	412	2
CagT	179	CagX	499	4
CagT	32	CagX	475	1
CagT	55	CagX	412	7
CagM	100	CagX	490	1
CagM	291	CagX	365	1
CagM	215	CagX	328	4
CagM	215	CagX	337	4
CagM	215	CagX	365	2
CagM	291	CagX	365	16
CagM	326	CagX	337	2
CagM	215	CagX	337	1
CagM	281	CagX	365	2
CagT	194	CagY	1898	2
CagT	78	CagY	1898	2
CagT	259	CagY	1924	1
CagT	194	CagY	1898	3
CagT	200	CagY	1924	2
CagT	200	CagY	1910	2
CagT	71	CagY	1910	4

CagT	101	CagY	1924	1
CagT	189	CagY	1898	2
CagT	252	CagY	1924	1
CagT	179	CagY	1924	1
CagT	179	CagY	1898	2
CagT	189	CagY	1910	2
CagT	189	CagY	1906	2
CagA	1147	CagF	124	2
CagA	1147	CagF	114	2
CagA	1147	CagF	136	3
CagA	137	CagF	124	1
CagA	1134	CagF	124	2
CagA	1175	CagF	124	4
CagA	1144	CagF	124	2
CagA	1180	CagF	124	2
CagA	518	CagF	124	2
CagA	1134	CagF	114	1
CagA	1175	CagF	114	6
CagA	1144	CagF	114	2
CagA	1180	CagF	114	3
CagA	518	CagF	114	2
CagA	1134	CagF	136	5
CagA	1175	CagF	136	2
CagA	1138	CagF	136	2
CagA	518	CagF	136	4
CagA	1083	CagF	9	3
CagA	587	CagF	256	2
CagA	592	CagF	256	2
CagA	577	CagF	256	2
CagA	580	CagF	256	9
CagA	646	CagF	256	2
CagA	644	CagF	256	2
CagA	784	Cag3	329	1
CagA	1091	CagX	337	4
CagA	1134	CagX	337	1
CagA	1083	CagX	337	2
CagA	646	CagX	337	1
CagM	236	CagY	1396	1
CagM	100	CagY	1924	1
CagM	230	CagY	1924	1
CagM	215	CagY	1924	2

CagM	124	CagY	95/225[b]	2
CagM	344	CagY	1631	2
CagM	198	CagY	1924	2
CagM	359	CagY	1924	2
CagM	297	CagY	1924	1
CagM	297	CagY	1646	2
CagM	291	CagY	1631	2
CagM	291	CagY	1924	1
CagM	291	CagY	1638	2
CagM	291	CagY	1646	2
CagM	291	CagY	1648	1
CagM	100	CagY	1910	3
CagM	344	CagY	1628	4
CagM	344	CagY	1631	3
CagM	344	CagY	1638	4
CagM	344	CagY	1924	2
CagM	344	CagY	1651	1
CagM	344	CagY	1617	3
CagM	344	CagY	1631	4
CagM	344	CagY	1646	4
CagM	344	CagY	1623	4
CagM	344	CagY	365	1
CagM	90	Cag3	305	3
CagM	77	Cag3	284	1
CagM	90	Cag3	284	1
CagM	40	Cag3	284	1
CagM	100	Cag3	284	2
CagM	105	Cag3	284	1
CagM	162	Cag3	282	1
CagM	124	Cag3	305	2
CagM	77	Cag3	259	1
CagM	170	Cag3	259	2
CagM	90	Cag3	259	2
CagM	95	Cag3	259	2
CagM	40	Cag3	259	2
CagM	100	Cag3	259	2
CagM	49	Cag3	259	2
CagM	105	Cag3	259	2
CagM	162	Cag3	282	1
CagM	141	Cag3	305	2
CagT	147	Cag3	162	2

CagT	92	Cag3	162	5
CagT	57	Cag3	73	1
CagT	55	Cag3	73	2
CagT	194	Cag3	101	2
CagT	57	Cag3	101	3

[a]Data in table is schematically depicted in Fig. 6B.

[b]Indicates two potential sites of crosslinks within the N-terminal repeat regions of CagY (not included in Fig. 7).

Table S3. Interprotein crosslinks detected in BS3-treated CagX Δ131-260 OMCCs[a].

Protein 1	Protein 1 lysine position	Protein 2	Protein 2 lysine position	Total BS3 interprotein crosslinks
CagY	1671	CagX	515	2
CagY	1628	CagX	365	3
CagY	1631	CagX	365	1
CagY	1924	CagX	393	4
CagY	1910	CagX	393	2
CagY	1906	CagX	393	2
CagY	1827	CagX	490	1
CagY	1910	CagX	490	2
CagY	1614	CagX	337	1
CagY	1593	CagX	337	1
CagY	1734	CagX	499	3
CagY	95/225[b]	CagX	127	1
CagY	1617	CagX	341	2
CagY	1737	CagX	499	2
CagY	1668	CagX	515	3
CagY	1671	CagX	515	4
CagY	1608	CagX	337	2
CagY	1612	CagX	337	2
CagY	1471	CagX	299	3
CagY	1465	CagX	299	1
CagY	1582	CagX	337	1
CagY	1465	CagX	53	4
CagY	1894	CagX	515	4
CagY	1910	CagX	372	1
CagY	1898	CagX	490	2
CagY	1898	CagX	374	1
CagY	1910	CagX	499	2
CagY	1898	CagX	504	2
CagY	1910	CagX	504	2
CagY	1898	CagX	409	1
CagY	1685	CagX	393	2
CagY	1685	CagX	490	1
CagY	1685	CagX	499	2
CagY	1685	CagX	409	2
CagY	1685	CagX	475	1
CagY	1617	CagX	337	3
CagY	1910	CagX	374	2

CagY	1471	CagX	40	1
CagY	1827	CagX	412	2
CagY	1910	CagX	412	1
CagY	1746	CagX	412	2
CagY	1906	CagX	490	3
CagY	1906	CagX	515	1
CagY	1906	CagX	499	1
CagY	1538	CagX	299	1
CagY	1598	CagX	287	2
CagY	1465	CagX	40	4
CagY	1924	CagX	365	2
CagY	1898	CagX	365	1
CagY	1910	CagX	365	2
CagY	1746	CagX	393	4
CagY	1746	CagX	490	2
CagY	1746	CagX	499	3
CagY	1746	CagX	409	2
CagY	1746	CagX	475	2
CagM	236	CagX	515	2
CagM	100	CagX	393	1
CagM	265	CagX	393	2
CagM	100	CagX	490	3
CagM	291	CagX	365	3
CagM	100	CagX	515	2
CagM	215	CagX	328	4
CagM	215	CagX	515	1
CagM	215	CagX	372	1
CagM	215	CagX	337	5
CagM	215	CagX	365	3
CagM	49	CagX	490	2
CagM	198	CagX	490	2
CagM	198	CagX	515	2
CagM	198	CagX	499	1
CagM	198	CagX	504	3
CagM	198	CagX	409	2
CagM	198	CagX	475	2
CagM	359	CagX	40	3
CagM	265	CagX	515	2
CagM	265	CagX	499	1
CagM	291	CagX	365	11
CagM	100	CagX	504	1

CagM	100	CagX	90	1
CagM	100	CagX	409	3
CagM	326	CagX	337	1
CagM	90	CagX	412	1
CagM	100	CagX	412	2
CagM	344	CagX	393	2
CagM	344	CagX	490	2
CagM	344	CagX	299	1
CagM	344	CagX	372	1
CagM	344	CagX	374	1
CagM	344	CagX	40	2
CagM	344	CagX	365	2
CagM	297	CagX	365	2
CagM	281	CagX	365	2
CagT	194	CagX	515	1
CagA	1091	CagF	9	2
CagA	1147	CagF	124	2
CagA	1147	CagF	114	2
CagA	1147	CagF	136	2
CagA	631	CagF	245	1
CagA	137	CagF	124	1
CagA	1134	CagF	124	2
CagA	1175	CagF	124	4
CagA	1138	CagF	124	2
CagA	1185	CagF	124	2
CagA	1144	CagF	124	1
CagA	1180	CagF	124	2
CagA	518	CagF	124	2
CagA	1175	CagF	114	5
CagA	1144	CagF	114	2
CagA	1180	CagF	114	2
CagA	518	CagF	114	2
CagA	137	CagF	136	1
CagA	1134	CagF	136	4
CagA	1175	CagF	136	2
CagA	1180	CagF	136	2
CagA	518	CagF	136	5
CagA	1083	CagF	9	3
CagA	522	CagF	136	2
CagA	587	CagF	256	2
CagA	631	CagF	256	2

CagA	592	CagF	256	1
CagA	577	CagF	256	2
CagA	580	CagF	256	8
CagA	646	CagF	256	2
CagA	644	CagF	256	1
CagA	1091	CagX	337	1
CagA	137	CagX	337	1
CagA	1083	CagX	337	1
CagA	704	CagM	230	1
CagA	715/760[b]	CagM	230	1
CagA	784	Cag3	329	2
CagM	124	CagY	95/225[b]	3
CagM	198	CagY	1924	1
CagM	198	CagY	95/225[b]	2
CagM	198	CagY	1827	1
CagM	198	CagY	1898	2
CagM	198	CagY	1646	3
CagM	198	CagY	1910	2
CagM	198	CagY	1906	2
CagM	198	CagY	1746	1
CagM	359	CagY	1631	2
CagM	359	CagY	1646	2
CagM	359	CagY	1623	1
CagM	100	CagY	1827	2
CagM	265	CagY	1827	2
CagM	100	CagY	1898	2
CagM	265	CagY	1898	1
CagM	297	CagY	1910	1
CagM	100	CagY	1685	1
CagM	100	CagY	1910	3
CagM	344	CagY	1628	2
CagM	344	CagY	1631	2
CagM	344	CagY	1638	5
CagM	344	CagY	1924	1
CagM	344	CagY	1617	2
CagM	344	CagY	1631	1
CagM	344	CagY	1646	4
CagM	344	CagY	1623	2
CagM	344	CagY	1465	1
CagM	371	CagY	1465	1
CagT	92	Cag3	162	1

[a]Data in table is schematically depicted in Fig. 6C.

[b]Indicates two potential sites of crosslinks within the C-terminus of CagA or the N-terminal repeat regions of CagY (not included in Fig. 7).

Table S4. Intraprotein crosslinks within 30 Å involving the CagX PI region from BS3-treated wild-type OMCCs[a].

CagX lysine 1	CagX lysine 1 domain	CagX lysine 2	CagX lysine 2 domain	Total BS3 intraprotein crosslinks[a]	Calculated distance (Å)[b]
251	PI	258	PI	20	17.9
255	PI	251	PI	12	10.3
255	PI	258	PI	12	8.9
130	PR	258	PI	11	20.0
255	PI	130	PR	11	27.8
130	PR	251	PI	10	25.9
255	PI	245	PI	10	18.5
155	PI	163	PI	8	12.3
127	PR	251	PI	7	17.7
130	PR	249	PI	7	23.7
245	PI	249	PI	7	6.1
230	PI	155	PI	6	17.7
245	PI	251	PI	6	9.8
155	PI	150	PI	5	8.6
155	PI	145	PI	5	15.3
155	PI	238	PI	5	23.0
238	PI	251	PI	5	19.7
245	PI	258	PI	5	26.4
249	PI	251	PI	5	5.4
258	PI	261	PR	5	8.9
238	PI	249	PI	4	16.1
255	PI	127	PR	4	22.5
127	PR	249	PI	3	22.1
103	PR	258	PI	2	25.3
127	PR	238	PI	2	30.2
127	PR	258	PI	2	24.1
130	PR	238	PI	2	29.5
155	PI	245	PI	2	29.4
155	PI	237	PI	2	19.5

197	PI	204	PI	2	10.9
230	PI	150	PI	2	23.9
230	PI	163	PI	2	21
237	PI	251	PI	2	21
238	PI	150	PI	2	25.3
238	PI	145	PI	2	26.4
245	PI	150	PI	2	28.9
245	PI	130	PR	2	23.6
255	PI	238	PI	2	28.6

[a]Table shows intraprotein crosslinks involving the PI domain that were detected at least twice.

[b]Distance between each lysine pair was calculated using the AlphaFold 3 model of the CagX PI (Fig. 4C).
